# CAND1 regulates lunapark for the proper tubular network of the endoplasmic reticulum

**DOI:** 10.1038/s41598-019-49542-x

**Published:** 2019-09-11

**Authors:** Hiroaki Kajiho, Yasunori Yamamoto, Toshiaki Sakisaka

**Affiliations:** Division of Membrane Dynamics, Department of Physiology and Cell Biology, Kobe University Graduate School of Medicine, Kobe, 650-0017 Japan

**Keywords:** Biochemistry, Endoplasmic reticulum

## Abstract

Endoplasmic reticulum (ER) tubules connect each other by three-way junctions, resulting in a tubular ER network. Oligomerization of three-way junction protein lunapark (Lnp) is important for its localization and the three-way junction stability. On the other hand, Lnp has an N-terminal ubiquitin ligase activity domain, which is also important for the three-way junction localization. To understand the mode of action of Lnp, we isolated Cullin-associated and neddylation-dissociated 1 (CAND1), a regulator of Skp1-Cul1-F-box (SCF) ubiquitin ligase, as a Lnp-binding protein by affinity chromatography. CAND1 and Lnp form a higher-molecular-weight complex *in vitro*, while they do not co-localize at the three-way junctions. CAND1 reduces the auto-ubiquitination activity of Lnp. CAND1 knockdown enhances proteasomal degradation of Lnp and reduces the tubular ER network in mammalian cells. These results suggest that CAND1 has the potency to promote the formation of the higher-molecular-weight complex with Lnp and reduce the auto-ubiquitination activity of Lnp, thereby regulating the three-way junction stability of the tubular ER network.

## Introduction

The endoplasmic reticulum (ER) is the largest organelle in eukaryotic cells and plays essential roles in various cellular functions including protein synthesis, transport and quality control, lipid synthesis, calcium storage, and detoxification of harmful substances^1,2^. The ER is a continuous membrane system composed of interconnected tubules and sheets^3,4^. The ER tubules and the edges of the ER sheets are characterized by relatively high membrane curvature. These high-curvature membranes are shaped by the two distinct classes of the ER membrane-shaping proteins conserved from yeasts to mammals, reticulons (Rtns) and REEPs/DP1/Yop1^5,6^. Rtns and REEPs/DP1/Yop1 insert their wedge-like transmembrane domains into the outer leaflet of the ER membrane and thereby expand the area of the outer leaflet relative to the inner leaflet, leading to generation and stabilization of the high membrane curvature of the ER tubules and the edges of the ER sheets^7–10^. In mammals, Arl6IP1, the ER membrane protein having the Rtn-like transmembrane domains, also shapes the ER tubules and the edges of the ER sheets in the same manner as Rtns and REEPs/DP1/Yop1^11^. On the other hand, flat areas of the ER sheets are shaped by CLIMP-63, another class of the ER membrane-shaping proteins^4,12^. Upon oligomerization, CLIMP-63 forms bridges across the luminal space of the ER and thereby flattens the ER membrane, leading to generation and stabilization of flat areas of the ER sheets^13,14^.

ER tubules are interconnected by three-way junctions into a reticular network. The three-way junctions are generated by homotypic membrane fusion between ER tubules in which atlastins (ATLs), dynamin-like GTPases anchored on the ER membrane, play key roles^15–18^. ATLs form a bridge between the tip of an ER tubule and the side of another one and catalyze membrane fusion in a GTP hydrolysis-dependent manner^19–21^, thereby generating a new three-way junction^22^. Importantly, a recent study has reconstituted the tubular ER network with synthetic liposomes and the purified recombinant proteins of ATLs and Rtns or REEPs *in vitro*^23^, indicating that these components constitute minimum requirements for formation of the tubular ER network. On the other hand, the tubular ER network as formed by ATLs and Rtns or REEPs is not static but dynamic *in vivo*^24–26^. Evidence is accumulating that the tubular ER network undergoes constant remodeling through elongation and retraction of tubules, sliding of the three-way junctions and ring closure (contraction of ER polygon rings)^27,28^. However, it remains unclear how the dynamics of the tubular ER network is regulated.

Several studies have suggested that stability of the three-way junctions is associated with regulation of the dynamics of the tubular ER network^27,29^. Lunapark (Lnp) is a conserved ER membrane protein composed of an N-terminal myristoylation motif, a short cytoplasmic domain, two transmembrane domains which adopt a hairpin configuration, and a long C-terminal cytoplasmic domain containing a coiled-coil domain, a zinc finger motif and the following LNPARK amino acids^30,31^, and has been shown to be a key molecule that stabilizes the three-way junctions^22,27,29^. The morphology of the three-way junctions is characterized by small, triangular sheets with concave edges^29^. Lnp preferentially sits on the concave edges of the three-way junctions, thereby stabilizing the negative curvature of the three-way junctions^27,29^. Importantly, knockout of Lnp not only decreases the number of the three-way junctions but also causes the ER tubule-to-sheet conversion^22^, suggesting that Lnp will regulate the dynamics of the tubular ER network through the three-way junction stability. Previous studies have demonstrated that Lnp has an ability to dimerize through the region encompassing the zinc finger motif *in vitro*^22,32^, and the Lnp mutant lacking this region abolishes localization to the three-way junctions^22^, indicating, upon oligomerization, Lnp localizes to and stabilizes the three-way junctions. Furthermore, phosphorylation of Lnp negatively regulates dimerization, leading to remodeling of the tubular ER network during mitosis^22^. On the other hand, it still remains to be clarified how assembly of Lnp into the dimer or possibly higher-order oligomers is positively regulated. Given that many studies have demonstrated that artificial manipulation of the protein level of Lnp by overexpression, knockdown or knockout induces severe morphological changes of the ER^22,27,29,30^, in addition to oligomerization, protein abundance of Lnp might be another key element for regulation of the tubular ER network. A recent study has shown that the short N-terminal cytoplasmic domain of Lnp unexpectedly has a ubiquitin ligase activity and is important for localization to the three-way junctions^33^. Interestingly, the ubiquitin ligase activity of Lnp is not associated with degradation of misfolded ER proteins as mediated by the ER-associated protein degradation pathway^33^, raising an attractive possibility that Lnp might ubiquitinate itself to control its abundance through the proteasomal degradation. However, it remains unknown whether the ubiquitin ligase activity of Lnp is indeed associated with control of its abundance for regulation of the tubular ER network.

In this study, we identified Cullin-associated and neddylation-dissociated 1 (CAND1)^34–39^, a regulator of Skp1-Cul1-F-box (SCF) ubiquitin ligase, as a Lnp-binding protein. We demonstrate that CAND1 has the potency to promote the formation of the higher-molecular-weight complex with Lnp and reduce the auto-ubiquitination activity of Lnp toward prevention of its proteasomal degradation, thereby regulating the tubular ER network. Our findings underscore that CAND1 is a positive regulator of Lnp and imply an unexpected link between the oligomerization and the ubiquitin ligase activity of Lnp for regulation of the tubular ER network.

## Results

### CAND1 is identified as a lunapark-binding protein

**Figure 1:**
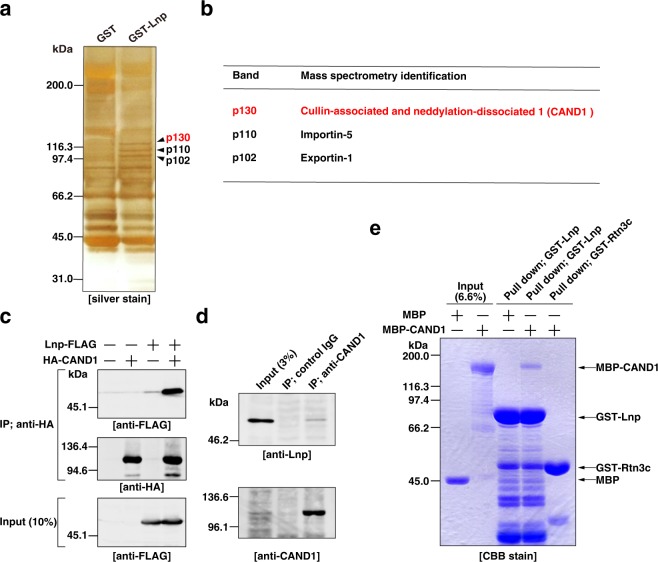
CAND1 is identified as a lunapark-binding protein. (**a**) Purification of Lnp-binding proteins. GST alone and GST-Lnp were immobilized on glutathione Sepharose and incubated with Triton X-100 extracts of *Sus scrofa* brains. The bound proteins were eluted with buffer containing 2M NaCl and subjected to SDS-PAGE followed by silver staining. The arrowheads indicate the three prominent bands (p130, p110, and p102) specifically detected in the GST-Lnp column. (**b**) Mass spectrometry identification of p130, p110, and p102. The three bands (p130, p110, and p102) in (**a**) were cut out and subjected to mass spectrometry analysis. The proteins with the highest Mascot score are shown. All the identified proteins are listed in Supplementary Table S1. (**c**) Co-immunoprecipitation of exogenous CAND1 and exogenous Lnp. HEK293 cells were transfected with the indicated combinations of Lnp-FLAG and HA-CAND1. NP-40 extracts of the transfected cells were immunoprecipitated with the anti-HA mAb, followed by immunoblotting with the anti-FLAG pAb and the anti-HA pAb. (**d**) Co-immunoprecipitation of endogenous CAND1 and endogenous Lnp. NP-40 extract of HeLa cells was immunoprecipitated with the mouse control IgG or the anti-CAND1 mAb, followed by immunoblotting with the anti-Lnp pAb and the anti-CAND1 mAb. (**e**) Direct binding between Lnp and CAND1 *in vitro*. GST-Lnp or GST-Rtn3c immobilized on glutathione Sepharose was incubated with MBP alone or MBP-CAND1. After being extensively washed, the bound proteins were subjected to SDS-PAGE followed by CBB staining.

We sought to identify a protein(s) that bound to Lnp by performing affinity chromatography. GST-fused Lnp (GST-Lnp) and GST alone were immobilized on glutathione Sepharose, respectively, and incubated with the Triton X-100 extracts of *Sus scrofa* brains. The bound proteins were eluted with buffer containing a high concentration of salt and subjected to SDS-PAGE followed by silver staining. Three bands at 130 kDa, 110 kDa, and 102 kDa were specifically detected in the GST-Lnp column (Fig. 1a). Mass spectrometry analysis identified these bands as CAND1, Importin-5, and Exportin-1, respectively (Fig. 1b). We listed all the proteins identified from the three bands in Supplementary Table S1. gp78, an E3 ubiquitin ligase involved in endoplasmic reticulum-associated degradation^40^, and ATLs have been reported to interact with Lnp^33,41^. The molecular weights of gp78 (~80 kDa) and ATLs (~60–70 kDa) are different from those of the three bands (130 kDa, 110 kDa, and 102 kDa). In agreement with this, gp78 and ATLs were not included in our list.

CAND1 is a cytosolic protein that regulates assembly of the SCF ubiquitin ligases in the ubiquitin-proteasome system^34–39^. Importin-5 and Exportin-1 are proteins that shuttle between the nucleus and the cytosol to mediate nucleocytoplasmic transport of proteins and RNAs through the nuclear pore complex^42,43^. Since Lnp is an ER-resident protein and does not shuttle between the nucleus and the cytosol, we focused on CAND1 in this study and characterized it in the context of a potential binding protein.

We assessed binding of CAND1 to Lnp by immunoprecipitation analysis. HA-tagged CAND1 (HA-CAND1) was transfected into HEK293 cells along with C-terminal FLAG-tagged Lnp (Lnp-FLAG), followed by immunoprecipitation with the anti-HA mAb. The samples were subjected to SDS-PAGE followed by immunoblotting with the anti-HA pAb and the anti-FLAG pAb. Lnp-FLAG was co-immunoprecipitated with HA-CAND1 (Fig. 1c). Endogenous CAND1 was immunoprecipitated with the anti-CAND1 mAb from the NP-40 extract of HeLa cells. The samples were subjected to SDS-PAGE followed by immunoblotting with the anti-CAND1 mAb and the anti-Lnp pAb. Endogenous Lnp was co-immunoprecipitated with endogenous CAND1 (Fig. 1d). These results validated that CAND1 binds to Lnp *in vitro*.

We next examined whether CAND1 directly binds to Lnp with the purified recombinant proteins. GST-Lnp and GST-Rtn3c, as a negative control, were immobilized on glutathione Sepharose and incubated with MBP-fused CAND1 (MBP-CAND1). After extensive wash of the resins, the bound proteins were subjected to SDS-PAGE followed by Coomassie brilliant blue (CBB) staining. MBP-CAND1 was pulled down with GST-Lnp (Fig. 1e). On the other hand, MBP-CAND1 was not pulled down with GST-Rtn3c. We also performed a reciprocal pull-down experiment in which MBP-CAND1 immobilized to amylose resin was incubated with GST-Lnp or GST-Rtn3c. As expected, GST-Lnp was pulled down with MBP-CAND1 (Supplementary Fig. S1). On the other hand, GST-Rtn3c was not pulled down with MBP-CAND1. These results indicate that CAND1 directly and specifically binds to Lnp *in vitro*.

We sought to identify the domains in Lnp responsible for the binding to CAND1. We generated several Lnp deletion mutants lacking the N-terminal ubiquitin ligase domain (ΔUb), the coiled-coil domain (ΔCC), and the zinc finger motif and the following LNPARK amino acids (ΔZn + LNPARK) (Supplementary Fig. S2a). In addition, we generated Lnp ΔTM by substituting the transmembrane domains with a polypeptide linker (GGS)_3_ to avoid aggregation, as reported previously^33^. Wild type (WT) and the deletion mutants of hexa histidine-tagged Lnp (His-Lnp) were immobilized on Ni-agarose and incubated with MBP-CAND1. After extensive wash of the resins, the bound proteins were subjected to SDS-PAGE followed by CBB staining. The binding of MBP-CAND1 to His-Lnp ΔZn + LNPARK was detected similarly to WT (Supplementary Fig. S2b). On the other hand, the binding of MBP-CAND1 to His-Lnp ΔUb, ΔTM, or ΔCC was reduced compared to WT, indicating these domains are important for the binding to CAND1.

We examined the co-localization of Lnp and CAND1 by observing the localization of mCherry-tagged Lnp (Lnp-mChe) and endogenous CAND1 in COS-7 cells. Lnp-mChe localized at the three-way junctions. On the other hand, endogenous CAND1 localized mainly in the nucleus and slightly in the cytoplasm (Supplementary Fig. S3). We could not observe that Lnp and CAND1 co-localize at the three-way junctions.

We examined whether Lnp, CAND1, and gp78 form a ternary complex. Lnp-FLAG, HA-CAND1, and gp78-myc were transfected into HEK293 cells, followed by immunoprecipitation with the anti-myc mAb. Lnp-FLAG was co-immunoprecipitated with gp78-myc (Supplementary Fig. S4a). On the other hand, HA-CAND1 was not co-immunoprecipitated with gp78-myc. We also performed a reciprocal immunoprecipitation with the anti-HA mAb. Lnp-FLAG was co-immunoprecipitated with HA-CAND1 (Supplementary Fig. S4b). On the other hand, gp78-myc was not co-immunoprecipitated with HA-CAND1. These results indicate that Lnp, CAND1, and gp78 do not form a ternary complex and suggest that the Lnp-CAND1 and the Lnp-gp78 complex are formed individually.

### CAND1 and lunapark form a higher-molecular-weight complex *in vitro*

**Figure 2:**
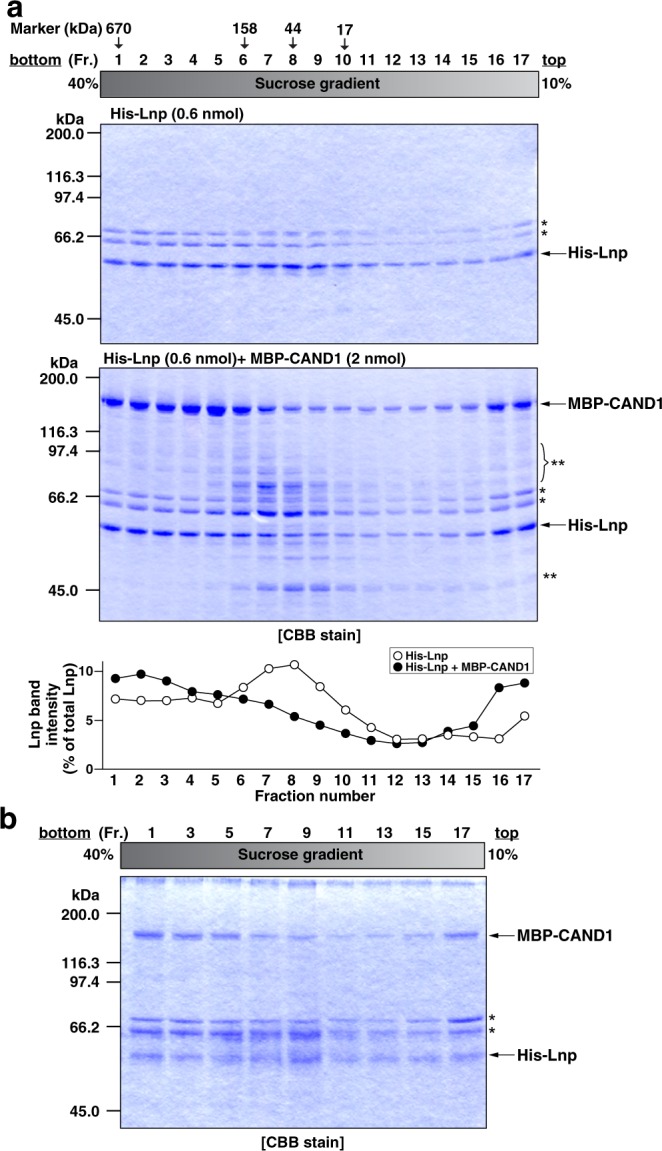
CAND1 and lunapark form a higher-molecular-weight complex *in vitro*. (**a**) The formation of a higher-molecular-weight complex between CAND1 and Lnp *in vitro*. His-Lnp (0.6 nmol) alone or the mixture of His-Lnp (0.6 nmol) and MBP-CAND1 (2 nmol) was loaded on top of a 10–40% (w/v) linear sucrose gradient and subjected to ultracentrifugation. Seventeen fractions were collected from the bottom of the gradient. The fractions were subjected to SDS-PAGE followed by CBB staining. To estimate the molecular weights of the fractionated samples, the molecular-size marker proteins with 670 kDa, 158 kDa, 44 kDa, and 17 kDa were similarly subjected to the sucrose density gradient ultracentrifugation, and their peak fractions are indicated with arrows above the fraction numbers. The intensity of the band of His-Lnp in each fraction was expressed as a percentage of the total intensity of the bands of His-Lnp from all the fractions. Open circles, His-Lnp alone. Closed circles, the mixture of His-Lnp and MBP-CAND1. The single and double asterisks denote contaminating proteins derived from the purification of His-Lnp and MBP-CAND1, respectively. (**b**) Incorporation of CAND1 into the higher-molecular-weight complex *in vitro*. The odd-numbered fractions of the mixture of His-Lnp and MBP-CAND1 in (**a**) were incubated with Ni-agarose. After being extensively washed, the bound proteins were subjected to SDS-PAGE followed by CBB staining. The asterisks denote contaminating proteins derived from the purification of His-Lnp.

We examined whether CAND1 and Lnp form a higher-molecular-weight complex *in vitro*. His-Lnp alone or His-Lnp incubated with MBP-CAND1 were size-fractionated by sucrose density gradient centrifugation. The samples were subjected to SDS-PAGE followed by CBB staining. His-Lnp alone was distributed from Fraction 1 through 9 with a single peak at Fraction 8 (Fig. 2a). The molecular weight marker with approximately 44 kDa was recovered in the Fraction 8 on a parallel gradient. These results indicate that His-Lnp predominantly exists as a monomer and that certain amount of His-Lnp exists as an oligomer. By contrast, His-Lnp incubated with MBP-CAND1 has a peak at Fraction 1, where the molecular weight marker with approximately 670 kDa was recovered. These results indicate that CAND1 promotes formation of a higher-molecular-weight complex of Lnp *in vitro*.

We next examined whether the higher-molecular-weight complex was formed through the interaction between Lnp alone, or Lnp and CAND1. From the odd-numbered fractions, His-Lnp was pulled down with Ni-agarose, and the samples were subjected to SDS-PAGE followed by CBB staining. MBP-CAND1 was pulled down with His-Lnp in Fractions 1, 3, and 5 more than those in Fractions 7, 9, and 11 (Fig. 2b). These results indicate that the higher-molecular-weight complex does not consist of only Lnp, but of Lnp and CAND1.

### CAND1 promotes the interaction of the N- and C-terminal regions of Lnp *in vitro*

**Figure 3:**
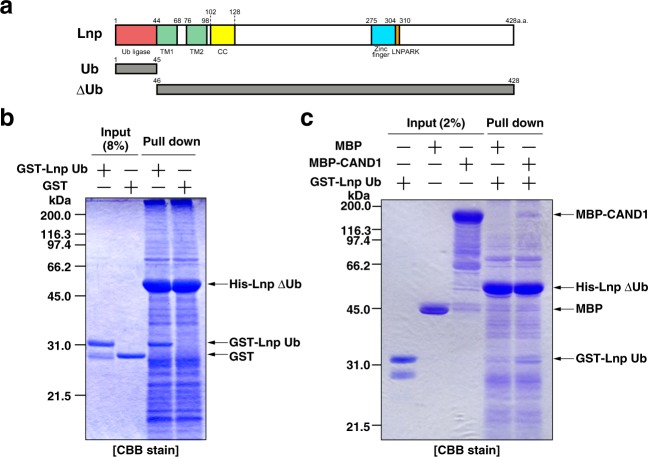
CAND1 promotes the interaction of the N- and C-terminal regions of lunapark *in vitro*. (**a**) Schematic representation of the primary structure of Lnp and the deletion mutants used in this study. Ub, ubiquitin. TM, transmembrane. CC, coiled-coil. (**b**) Direct binding between the N- and C-terminal regions of Lnp. His-Lnp ΔUb immobilized on Ni-agarose was incubated with GST-Lnp Ub or GST alone. After being extensively washed, the bound proteins were subjected to SDS-PAGE followed by CBB staining. (**c**) Increasing the direct binding between the N- and the C-terminal regions of Lnp by CAND1 *in vitro*. His-Lnp ΔUb immobilized on Ni-agarose was incubated with the indicated combinations of MBP alone, MBP-CAND1, or GST-Lnp Ub. After being extensively washed, the bound proteins were subjected to SDS-PAGE followed by CBB staining.

We examined whether the N-terminal region of Lnp binds to the C-terminal region. We generated the Lnp deletion mutant comprising the N-terminal ubiquitin ligase domain (Lnp Ub) (Fig. 3a). His-Lnp ΔUb immobilized on Ni-agarose was incubated with GST-Lnp Ub. After extensive wash of the resins, the bound proteins were subjected to SDS-PAGE followed by CBB staining. The binding of GST-Lnp Ub to His-Lnp ΔUb was detected (Fig. 3b), indicating that Lnp forms the inter-molecular interaction between the N- and C-terminal regions. To examine the effect of CAND1 on the inter-molecular interaction of Lnp, His-Lnp ΔUb immobilized on Ni-agarose was incubated with GST-Lnp Ub and MBP-CAND1. After extensive wash of the resins, the bound proteins were subjected to SDS-PAGE followed by CBB staining. The binding of GST-Lnp Ub to His-Lnp ΔUb was increased in the presence of MBP-CAND1 more than in the presence of MBP (Fig. 3c). The result suggests that CAND1 promotes the inter-molecular interaction of Lnp between the N- and C-terminal regions *in vitro*, leading to the formation of the higher-molecular-weight complex.

### CAND1 reduces the auto-ubiquitination activity of lunapark

**Figure 4:**
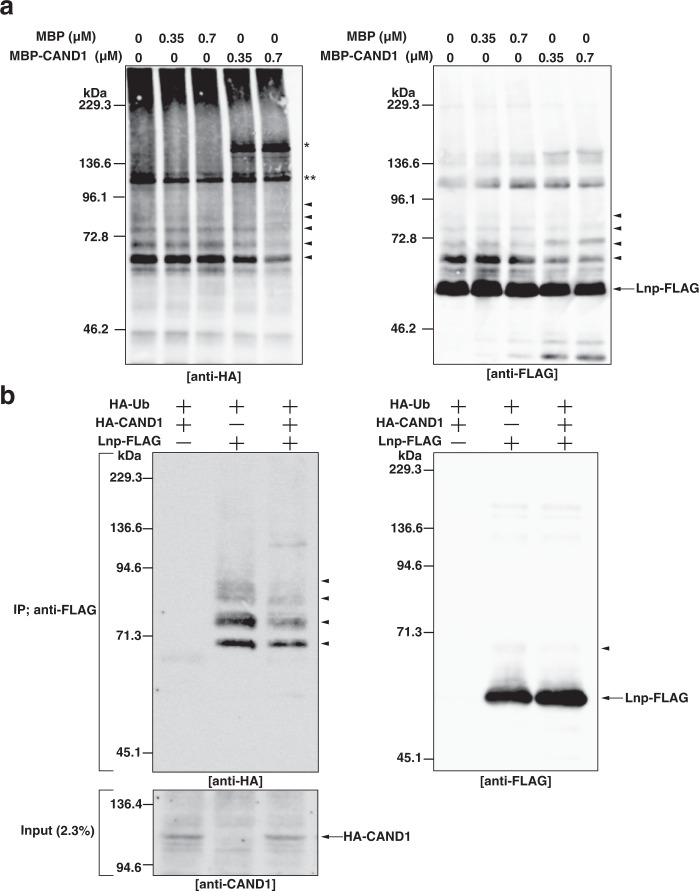
CAND1 reduces the auto-ubiquitination activity of lunapark. (**a**) Reduction of the auto-ubiquitination activity of Lnp by CAND1 *in vitro*. His-E1 (80 nM), His-UBE2D1 (4 µM), Lnp-FLAG (0.7 µM), HA-Ub (30 µM), and magnesium/ATP (2 mM) were incubated in the presence of MBP alone or MBP-CAND1 at the indicated concentrations at 37 °C for 150 min to allow auto-ubiquitination reactions to proceed. The samples were subjected to SDS-PAGE followed by immunoblotting with the anti-HA mAb and the anti-FLAG pAb. The arrowheads denote the high*-*molecular*-*weight Lnp-ubiquitin conjugates formed by auto-ubiquitination of Lnp-FLAG. The single asterisk denotes non-specific ubiquitination of MBP-CAND1. The double asterisk denotes the His-E1 bound to HA-Ub. (**b**) Reduction of the auto-ubiquitination activity of Lnp by CAND1 in mammalian cells. HEK293 cells were transfected with the indicated combinations of HA-Ub, HA-CAND1, and Lnp-FLAG. NP-40 extracts of the transfected cells were heated at 90 °C for 5 min in the presence of 1% SDS to allow Lnp-FLAG to denature and completely dissociate from the binding proteins. After being diluted for renaturation, free Lnp-FLAG was immunoprecipitated with the anti-FLAG mAb and subjected to SDS-PAGE followed by immunoblotting with the anti-HA pAb and the anti-FLAG pAb. To check the expression of HA-CAND1, the input sample was subjected to SDS-PAGE followed by immunoblotting with the anti-CAND1 mAb. The arrowheads denote the high*-*molecular*-*weight Lnp-ubiquitin conjugates formed by auto-ubiquitination of Lnp-FLAG.

A recent study has shown that Lnp has the ubiquitin ligase activity as detected by auto-ubiquitination^33^. Therefore, we examined the effect of CAND1 on auto-ubiquitination of Lnp. Lnp-FLAG was incubated with His-tagged recombinant ubiquitin-activating enzyme (E1), His-UBE2D1 as a ubiquitin-conjugating enzyme (E2), HA-tagged ubiquitin (HA-Ub), and magnesium/ATP in the presence of MBP or MBP-CAND1. In the presence of MBP, Lnp-FLAG was auto-ubiquitinated as judged by the ladder of high-molecular-weight Lnp-ubiquitin conjugates immunoreactive for both the HA and FLAG epitopes (Fig. 4a). In the presence of MBP-CAND1, the level of the high-molecular-weight Lnp-ubiquitin conjugates was decreased significantly.

We examined whether CAND1 reduces the ubiquitination activity of Lnp *in vitro* through the interaction between CAND1 and Lnp. We tried to generate the Lnp mutant that cannot bind to CAND1. Based on the binding assay (Supplementary Fig. S2b), we speculated that the N-terminal ubiquitin ligase domain of Lnp (Lnp Ub) could not bind to CAND1. GST-Lnp WT or GST-Lnp Ub immobilized on glutathione Sepharose was incubated with MBP-CAND1. After extensive wash of the resins, the bound proteins were subjected to SDS-PAGE followed by CBB staining. The binding of MBP-CAND1 to GST-Lnp Ub was not detected (Supplementary Fig. S5a**)**, whereas the binding of MBP-CAND1 to GST-Lnp WT was detected. These results indicate that the N-terminal ubiquitin ligase domain of Lnp does not bind to CAND1. A recent study has shown that the N-terminal ubiquitin ligase domain of Lnp fused to GST has an activity to synthesize ubiquitin chains^33^. To test the effect of CAND1 on the ubiquitination activity of the N-terminal ubiquitin ligase domain of Lnp, GST-Lnp Ub was incubated with MBP-CAND1, His-E1, His-UBE2D1, HA-Ub, and magnesium/ATP. MBP-CAND1 did not reduce the ubiquitin chains formed by GST-Lnp Ub (Supplementary Fig. S5b**)**, indicating that CAND1 does not affect the ubiquitin ligase activity of the N-terminal region of Lnp.

To validate the inhibitory effect of CAND1, we performed *in vivo* ubiquitination assay. Both Lnp-FLAG and HA-CAND1 or either of them were transfected into HEK293 cells along with HA-Ub. We incubated the cells with the proteasome inhibitor MG132 for 4 h to accumulate ubiquitinated proteins. The cells were lysed with 1% NP-40, and the cell lysates were incubated at 90 °C in the presence of 1% SDS for 5 min to allow Lnp-FLAG to denature and completely dissociate from the binding proteins including CAND1. Free Lnp-FLAG was then immunoprecipitated with the anti-FLAG mAb, followed by immunoblotting with the anti-HA pAb and the anti-FLAG pAb. The ladder of high*-*molecular-weight Lnp-ubiquitin conjugates immunoreactive for the HA epitope was detected in the absence of HA-CAND1 (Fig. 4b), indicating that Lnp-FLAG was auto-ubiquitinated in the HEK293 cells. It is noted that, unlike immunoblotting with the anti-HA pAb, it was hard to detect the high*-*molecular-weight Lnp-ubiquitin conjugates by immunoblotting with the anti-FLAG pAb. Importantly, the level of the Lnp-ubiquitin conjugates was significantly decreased in the presence of HA-CAND1. These results indicate that CAND1 reduces the auto-ubiquitination activity of Lnp in the mammalian cells.

Collectively, these results indicate that CAND1 has the ability to reduce the auto-ubiquitination activity of Lnp.

### Lysine 100 is one of the major ubiquitination sites of lunapark

**Figure 5:**
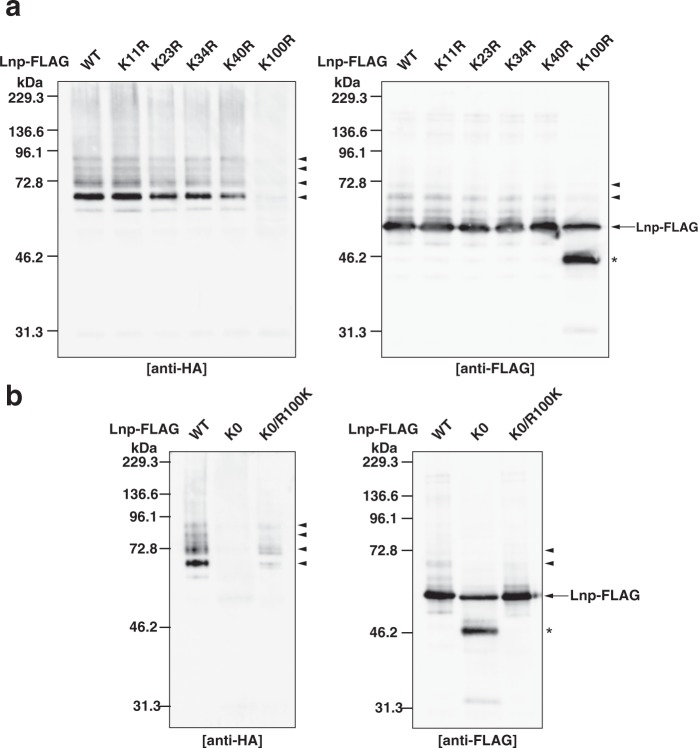
Lysine 100 is one of the major ubiquitination sites of lunapark. (**a**) Reduction of the auto-ubiquitination activity of the Lnp K100R mutant. HEK293 cells were transfected with wild type (WT) or the single-point mutants from lysine (K) to arginine (R) in the indicated amino acid positions of Lnp-FLAG along with HA-Ub. Lnp-FLAG was immunoprecipitated with the anti-FLAG mAb as in Fig. 4b, and subjected to SDS-PAGE followed by immunoblotting with the anti-HA pAb and the anti-FLAG pAb. The arrowheads denote the higher-molecular-weight Lnp-ubiquitin conjugates formed by auto-ubiquitination of Lnp-FLAG. The asterisk denotes degraded Lnp K100R-FLAG. (**b**) Auto-ubiquitination of lysine 100 of Lnp. HEK293 cells were transfected with WT, the mutant with all the lysine residues substituted with arginine (K0), or the one which contains only single lysine at amino acid position 100 with all other lysine residues substituted with arginine (K0/R100K) of Lnp-FLAG along with HA-Ub. Lnp-FLAG was immunoprecipitated with the anti-FLAG mAb as in Fig. 4b, and subjected to SDS-PAGE followed by immunoblotting with the anti-HA pAb and the anti-FLAG pAb. The arrowheads denote the high*-*molecular*-*weight Lnp-ubiquitin conjugates formed by auto-ubiquitination of Lnp-FLAG. The asterisk denotes degraded Lnp K0-FLAG.

We sought to identify the ubiquitination sites in Lnp. To determine the region required for the auto-ubiquitination of Lnp, we generated a set of C-terminal deletion mutants of Lnp. We performed *in vivo* ubiquitination assay by transfecting the FLAG-tagged Lnp mutants and HA-Ub into HEK293 cells. Among the mutants, the FLAG-tagged N-terminal 100 amino acids of Lnp (Lnp N100-FLAG) (Supplementary Fig. S6a) was sufficient to be auto-ubiquitinated as much as full-length Lnp judged by the ladder immunoreactive for the HA epitope (Supplementary Fig. S6b). The result indicates that the N-terminal 100 amino acids of Lnp contain the major ubiquitination sites. Generally, ubiquitin makes an isopeptide bond with a lysine on the target protein. Therefore, we generated a set of Lnp mutants in which individual lysine in the N-terminal 100 amino acids was substituted with arginine (Lnp K11R, K23R, K34R, K40R, and K100R). The ubiquitination activities of Lnp WT and the mutants were tested by *in vivo* ubiquitination assay. The ladders immunoreactive for the HA epitope detected in Lnp K11R, K23R, K34R, and K40R-FLAG were comparable to the ladder detected in Lnp WT-FLAG (Fig. 5a). By contrast, the intensity of the ladder detected in Lnp K100R-FLAG was reduced significantly. To confirm the expression levels of WT and the mutants of Lnp, the samples were immunoblotted with the anti-FLAG pAb. A single band corresponding to the expected molecular weight for full-length Lnp was detected in Lnp WT, K11R, K23R, K34R, and K40R-FLAG (Fig. 5a). By contrast, in addition to the band corresponding to the expected molecular weight for full-length Lnp, the lower band was detected in Lnp K100R-FLAG. The lower band might be a subpopulation of Lnp K100R-FLAG that undergoes degradation. We further generated the Lnp mutant with all the lysine residues substituted with arginine (Lnp K0), and the one which contains only single lysine at amino acid position 100 with all other lysine residues substituted with arginine (Lnp K0/R100K). The ubiquitination activities of WT and the mutants were tested by *in vivo* ubiquitination assay. Lnp K0-FLAG did not show any immunoreactivity for the HA epitope (Fig. 5b). By contrast, Lnp K0/R100K-FLAG showed the ladder immunoreactive for the HA epitope. Similar to Lnp K100R-FLAG, two bands immunoreactive for the FLAG epitope were detected in Lnp K0-FLAG (Fig. 5b). We cannot rule out the possibility that a subpopulation of Lnp K100R-FLAG and Lnp K0-FLAG undergoes degradation through protein misfolding, and results in losing the auto-ubiquitination activities. These results indicate that lysine 100 is one of the major ubiquitination sites of Lnp.

### CAND1 is involved in proteasomal degradation of lunapark

**Figure 6:**
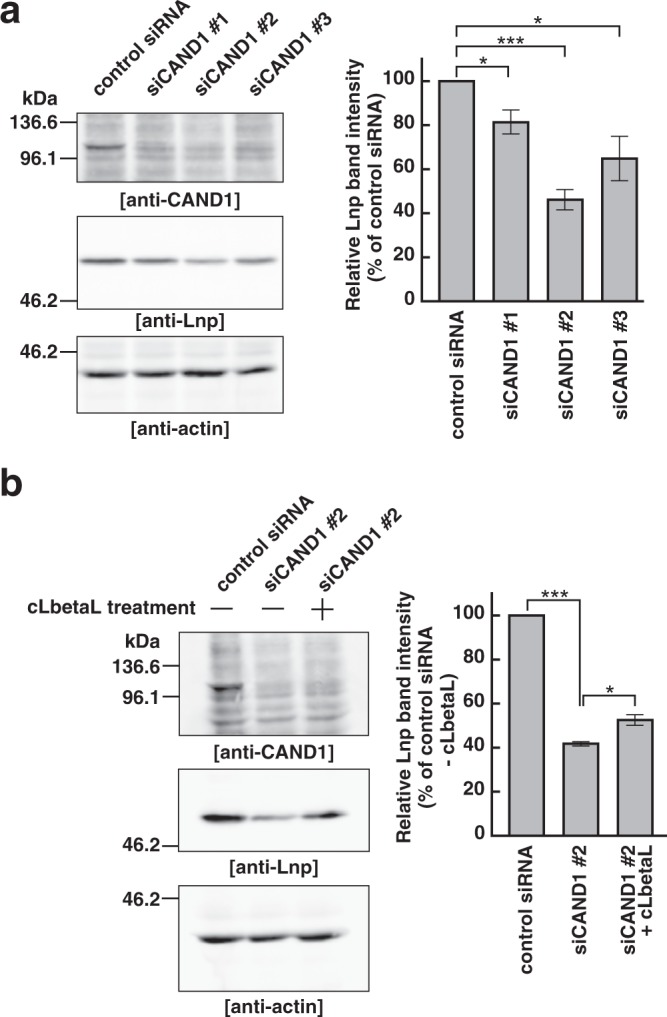
CAND1 regulates proteasomal degradation of lunapark. (**a**) Decrease of the protein level of endogenous Lnp in the CAND1 knocked-down cells. COS-7 cells were transfected with the control siRNA or three independent siRNAs targeting CAND1 (siCAND1 #1–3). The total cell extracts were subjected to SDS-PAGE followed by immunoblotting with the anti-CAND1 mAb, the anti-Lnp pAb, and the anti-actin mAb. The intensities of immunoreactive bands for Lnp were normalized to those for actin and expressed as percentages of the control siRNA-transfected cells in the right panel. Data are the averages ± SEM of three independent experiments. *P < 0.05, ***P < 0.001; paired Student’s *t*-test. (**b**) Restoration of the protein level of endogenous Lnp in the CAND1 knocked-down cells by a proteasome inhibitor. The COS-7 cells transfected with the control siRNA or siCAND1 #2 were cultured in the presence (+) or absence (−) of clasto-lactacystin β-lactone (cLbetaL) for 24 h. The total cell extracts were subjected to SDS-PAGE followed by immunoblotting with the anti-CAND1 mAb, the anti-Lnp pAb, and the anti-actin mAb. The intensities of immunoreactive bands for Lnp were normalized as in (**a**) and the relative band intensities are shown in the right panel. Data are the averages ± SEM of three independent experiments. *P < 0.05, ***P < 0.001; paired Student’s *t*-test.

We examined the effect of CAND1 knockdown on the protein level of endogenous Lnp. We reasoned that, if CAND1 reduced the auto-ubiquitination activity of endogenous Lnp, CAND1 knockdown would result in enhancement of proteasomal degradation of endogenous Lnp. To examine this reasoning, three independent siRNAs targeting CAND1 (siCAND1 #1, #2, and #3) were transfected into COS-7 cells and the cell lysates were subjected to SDS-PAGE followed by immunoblotting. These siRNAs successfully knocked down endogenous CAND1 as confirmed by immunoblotting with the anti-CAND1 mAb and the efficacy of siCAND1 #2 was the strongest (Fig. 6a). Importantly, in good agreement with the above reasoning, the protein levels of endogenous Lnp decreased in the CAND1 knocked-down cells relative to the control siRNA-transfected cells (Fig. 6a). Particularly, the siCAND1 #2-transfected cells showed the lowest protein level of Lnp (Fig. 6a), suggesting that the efficacy of the CAND1 siRNAs was correlated with decrease of protein levels of Lnp. We treated the siCAND1 #2-transfected cells with proteasome inhibitors, clasto-lactacystin β-lactone (cLbetaL) or MG132. cLbetaL and MG132 moderately restored the protein level of Lnp (Fig. 6b and Supplementary Fig. S7), indicating that the proteasomal degradation is involved in the decrease of protein levels of endogenous Lnp caused by CAND1 knockdown.

We examined whether the ubiquitination-deficient mutant of Lnp (Lnp K100R) is more resistant to the effects of CAND1 knockdown than WT. Lnp-mChe or Lnp K100R-mChe was co-transfected with siCAND1 #2 into COS-7 cells, and the cells were split into three dishes and cultured for 24, 48, or 72 h, respectively. The total cell lysates were subjected to SDS-PAGE followed by immunoblotting. CAND1 was detected at 24 h after transfection, and was efficiently knocked down at 72 h after transfection (Supplementary Fig. S8). The protein amount of Lnp-mChe at 72 h was reduced to 20% of that at 24 h. By contrast, the protein amount of Lnp K100R-mChe at 72 h was reduced to 67% of that at 24 h, suggesting that Lnp K100R is more resistant to the effects of CAND1 knockdown than WT.

These results indicate that CAND1 is involved in proteasomal degradation of Lnp, supporting that CAND1 reduces the auto-ubiquitination activity of Lnp in mammalian cells.

### CAND1 is involved in regulation of the tubular ER network

**Figure 7:**
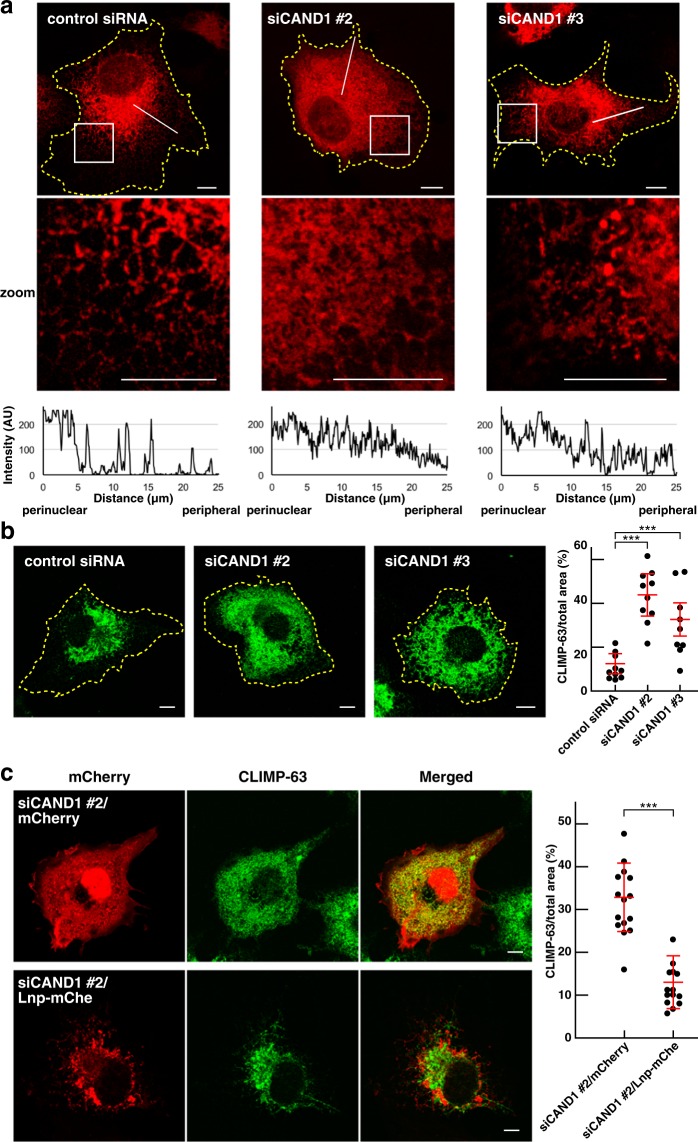
CAND1 is involved in regulation of the tubular ER network. (**a**) Alteration of the ER morphology in the CAND1 knocked-down cells. COS-7 cells were transfected with the control siRNA, siCAND1 #2, or siCAND1 #3, followed by transfection with the baculovirus carrying ER-RFP to label their entire ER structures. The yellow dashed lines indicate the edges of the cells. The boxed areas are enlarged to highlight the decrease of the reticular pattern of ER-RFP and the increase of the non-reticular pattern of ER-RFP in the peripheral area by CAND1 knockdown (zoom). The intensity profiles of ER-RFP on the lines are expressed as arbitrary units (AU) in the bottommost panels with directions from perinuclear to peripheral area. (**b**) Expansion of the ER sheets to the peripheral area in the CAND1 knocked-down cells. The COS-7 cells transfected with the control siRNA, siCAND1 #2, or siCAND1 #3 were immunostained with the anti-CLIMP-63 mAb. The yellow dashed lines indicate the edges of the cells. The ratios of CLIMP-63-staining area to total cell area are shown in the rightmost panel, in which each dot indicates the ratio in a single cell. N = 10. The averages ± SD are indicated by red lines. ***P < 0.001; paired Student’s *t*-test. (**c**) Restoration of ER morphology in the CAND1 knocked-down cells by exogenous Lnp expression. COS-7 cells were transfected with siCAND1 #2, followed by transfection with mCherry or mCherry-tagged Lnp (Lnp-mChe). The cells were immunostained with the anti-CLIMP-63 mAb. The ratios of CLIMP-63-staining area to total cell area are shown in the rightmost panel, in which each dot indicates the ratio in a single cell. N = 15. The averages ± SD are indicated by red lines. ***P < 0.001; paired Student’s *t*-test. Scale bars, 10 μm.

We examined the effect of CAND1 knockdown on the ER morphology. Lnp has been well characterized as a key molecule that stabilizes the three-way junctions^22,27,29^. Decreasing the protein level of Lnp has been shown to destabilize the three-way junctions, leading to alteration of the ER morphology^22,27,29^. Therefore, we reasoned that if CAND1 regulated the proteasomal degradation of functional Lnp but not non-functional misfolded Lnp, CAND1 knockdown would alter the ER morphology through destabilization of the three-way junctions. To examine this reasoning, the ER structures in the siRNA-transfected COS-7 cells were entirely labeled with ER-RFP, RFP fused to the ER signal sequence of calreticulin and KDEL (ER retention signal), by transfecting the recombinant baculovirus carrying ER-RFP. The control siRNA-transfected cells showed the reticular pattern of ER-RFP indicative of the normal tubular ER network in the peripheral area (Fig. 7a). By contrast, the CAND1 knocked-down cells significantly decreased the peripheral reticular pattern of ER-RFP relative to the control siRNA-transfected cells, and instead increased the non-reticular pattern of ER-RFP in the peripheral area. Besides, consistent with the efficacy of the siRNAs as shown in Fig. 6a, siCAND1 #2 decreased the peripheral reticular pattern of ER-RFP more than siCAND1 #3. These results indicate that, in good agreement with the above reasoning, CAND1 knockdown alters the ER morphology, in particular reduces the tubular ER network.

To further characterize the reduction of the tubular ER network caused by CAND1 knockdown, the CAND1 knocked-down cells were immunostained for CLIMP-63, an ER membrane protein that localizes at the ER sheets. Consistent with the previous studies^13,14^, CLIMP-63 localization was tightly restricted to the perinuclear area where the ER sheets were enriched in the control siRNA-transfected cells (Fig. 7b). By contrast, CLIMP-63 localization was aberrantly extended to the peripheral area in the CAND1 knocked-down cells. Quantification of the CLIMP-63 localization showed that the CAND1 knocked-down cells significantly increased the ratio of CLIMP-63 staining area/total area relative to the control siRNA-transfected cells. Besides, consistent with the efficacy of the siRNAs as shown in Fig. 6a, siCAND1 #2 increased the ratio of CLIMP-63 staining area/total more than siCAND1 #3. These results indicate that CAND1 knockdown causes expansion of the ER sheets to the peripheral area, thereby reducing the tubular ER network.

We examined whether the ER morphology altered by CAND1 knockdown is attributed to the dysfunction of other E3 ligases than Lnp. CAND1 regulates assembly of the SCF ubiquitin ligases^34–39^. siRNA-mediated silencing of Cul1 or Skp1, the essential components of the SCF ubiquitin ligase^44^, has been shown to reduce the ubiquitination of their target substrates^45,46^. siRNA targeting Cul1 or Skp1 (siCul1 or siSkp1) was transfected into COS-7 cells, and the siRNA-transfected cells were immunostained for CLIMP-63. siCul1 and siSkp1 efficiently knocked down the expression of Cul1 mRNA and Skp1 mRNA, respectively (Supplementary Fig. S9a). CLIMP-63 localization was restricted to perinuclear area in both siCul1- and siSkp1-transfected cells, similar to CLIMP-63 localization in the control siRNA-transfected cells (Supplementary Fig. S9b). These results suggest that the ER morphology altered by CAND1 knockdown is not attributed to the dysfunction of the SCF ubiquitin ligases.

To examine whether the reduction of the tubular ER network caused by CAND1 knockdown was attributed to decreased protein level of functional Lnp (i.e. destabilization of the three-way junctions), Lnp-mChe or mCherry alone was exogenously expressed in the siCAND1 #2-transfected cells, the CAND1 knocked-down cells having the most severe effect on the expansion of the ER sheets as shown in Fig. 7b. mCherry alone diffusely distributed throughout the cytoplasm whereas Lnp-mChe mainly localized at the three-way junctions of the ER (Fig. 7c), indicating that exogenously expressed Lnp-mChe was functional in the CAND1 knocked-down cells. Consistent with the results shown in Fig. 7b, CLIMP-63-positive ER sheets aberrantly expanded to the peripheral area in the CAND1 knocked-down cells expressing mCherry alone (Fig. 7c). By contrast, CLIMP-63-positive ER sheets were tightly restricted to the perinuclear area in the CAND1 knocked-down cells expressing Lnp-mChe, and instead the tubular ER network appeared in the peripheral area. The ratio of CLIMP-63 staining area/total area was decreased in the CAND1 knocked-down cells expressing Lnp-mChe relative to those expressing mCherry alone. We examined whether the ubiquitination-deficient mutant (Lnp K100R) rescued the expansion of the CLIMP-63-positive ER sheets in the CAND1 knocked-down cells. Similar to Lnp-mChe, Lnp K100R-mChe mainly localized to three-way junctions, and rescued the expansion of the CLIMP-63-positive ER sheets (Supplementary Fig. S10**)**. The fluorescence intensities of Lnp-mChe and Lnp K100R-mChe did not show significant difference. It is noted that, unlike Supplementary Fig. S8, COS-7 cells were transfected with siCAND1 #2, and cultured for 72 h. The siCAND1 #2-transfected cells were transfected with Lnp-mChe or Lnp K100R-mChe, and cultured for 24 h. It was technically difficult to achieve the same transfection efficiency between Lnp-mChe and Lnp K100R-mChe. Thus, we could not compare the effects of WT and K100R or show the difference between them. These results indicate that exogenous expression of functional Lnp rescues expansion of the ER sheets and reduction of the tubular ER network caused by CAND1 knockdown.

Collectively, these results indicate that CAND1 is involved in regulation of the tubular ER network by controlling the protein level of Lnp (i.e. by controlling the three-way junction stability).

## Discussion

In this study, we identified CAND1 as a protein that directly binds to Lnp. CAND1 and Lnp formed a higher-molecular-weight complex. CAND1 reduced the auto-ubiquitination activity of Lnp, thereby regulating the three-way junction stability of the tubular ER network. While these results indicate that CAND1 is a positive regulator of Lnp, they also raise the possibility that the status of the higher-molecular-weight complex might be associated with regulation of the ubiquitin ligase activity. Indeed, given that Lnp mostly existed as the higher-molecular-weight complex in the presence of CAND1 *in vitro* as shown in Fig. 2, it seems plausible that Lnp would inactivate its ubiquitin ligase activity by oligomerization rather than CAND1 binding. However, the mechanistic insights into how CAND1 promotes oligomerization of Lnp through direct binding and how the oligomerization inactivates the ubiquitin ligase activity still remain unknown. We showed that CAND1 increased the binding of the N-terminal region of Lnp to the C-terminal region (Fig. 3c). These results indicate that CAND1 promotes the inter-molecular interaction of Lnp between the N- and C-terminal regions, leading to the formation of the higher-molecular complex. On the other hand, several studies have demonstrated that Lnp forms a dimer through the C-terminal region encompassing the zinc finger motif ^22,32^. In addition, another study has shown that the short N-terminal cytoplasmic domain encompassing the ubiquitin ligase activity also forms a dimer *in vitro*^33^. Further biochemical and structural studies of the higher-molecular-weight complex of CAND1 and Lnp will be required to address these concerns.

We have shown that CAND1 binds to Lnp *in vitro* (Fig. 1) but not that CAND1 and Lnp co-localize at the three-way junctions (Supplementary Fig. S3), raising the possibility that CAND1 does not bind to Lnp in cells. Nevertheless, CAND1 knockdown decreased the amount of endogenous Lnp (Fig. 6a), and expanded the ER sheets and reduced the tubular ER network (Fig. 7a,b). Exogenous expression of Lnp rescued the altered ER morphology (Fig. 7c). These results suggest that CAND1 regulates the three-way junctions upstream of Lnp.

Since oligomerization of Lnp has been shown to be required for its localization to the three-way junctions^22^, CAND1 will be likely to regulate the localization of Lnp. On the other hand, CAND1 also regulates the protein abundance of Lnp through prevention of its proteasomal degradation as shown in Fig. 6. CAND1 has been shown to undergo cycles of binding to and dissociation from the SCF ubiquitin ligase, resulting in promoting exchange of substrates. Analogously, one possible explanation as to how the activities of CAND1 for Lnp are coupled for stabilization of the three-way junctions would be as follows. CAND1 promotes the inter-molecular interaction of Lnp between the N- and C-terminal region, leading to the formation of a Lnp dimer. CAND1 is dissociated from the Lnp dimer. CAND1 undergoes cycles of binding to and dissociation from Lnp, resulting in oligomerization of Lnp. Eventually, the Lnp oligomer would be concentrated to the three-way junctions. Consistent with the idea, CAND1 is not concentrated at the three-way junctions (Supplementary Fig. S3).

We showed that CAND1 forms a higher-molecular-weight complex with Lnp *in vitro* by using sucrose density gradient centrifugation (Fig. 2). On the other hand, we have not shown that the oligomerized Lnp and CAND1 co-localized at the three-way junctions of the tubular ER network in cells (Supplementary Fig. S3). The discrepancy between them suggests that there should be a molecular mechanism by which CAND1 binding to oligomerized Lnp at the three-way junctions is actively dissociated from oligomerized Lnp in cells. On the other hand, we cannot rule out the possibility that the higher-molecular-weight complex of Lnp and CAND1 is distinct from oligomerized Lnp responsible for its association with the three-way junctions. Our results are insufficient to conclude that the effects of CAND1 on Lnp are mediated by promotion of oligomerization. Further studies are required to elucidate these points.

The tubular ER network has been shown to be constantly remodeled through elongation and retraction of tubules, sliding of the three-way junctions and ring closure^27,28^. Evidence is accumulating that stabilization of the three-way junctions by Lnp is associated with maintenance and remodeling of the tubular ER network^22,27,29^. Our results in this study indicate that CAND1 is a positive regulator of Lnp toward stabilization of the three-way junctions. However, in addition to the positive regulation of Lnp by CAND1, negative regulation of Lnp toward destabilization of the three-way junctions will be required for remodeling of the tubular ER network. The recent study has shown that phosphorylation of Lnp inactivates the Lnp function by decreasing dimerization, leading to destabilization of the three-way junctions^22^. Therefore, one possibility is that the Lnp phosphorylation might cause disassembly of the higher-order oligomers of Lnp as formed by CAND1 into monomers at the three-way junctions. The phosphorylated monomers might be preferentially auto-ubiquitinated and promptly removed through the proteasomal degradation as described above, leading to destabilization of the three-way junctions. In this scenario, the protein level of the functional higher-order oligomers of Lnp at the three-way junctions, i.e. stability of the three-way junctions, would be balanced by CAND1 and phosphorylation, which in turn would underlie maintenance and remodeling of the tubular ER network. Therefore, the expansion of the ER sheets and the reduction of the tubular ER network in the CAND1 knocked-down cells as shown in Fig. 7 might be attributed to destabilization of the three-way junctions caused by disbalance of CAND1 and phosphorylation. In cells having the extensive tubular ER network, oligomerization of Lnp promoted by CAND1 might be dominant relative to disassembly of Lnp oligomers by phosphorylation, resulting in the reduction of the auto-ubiquitination activity. Consistent with the idea, the auto-ubiquitination activity of the overexpressed Lnp was weak (Figs 4b and 5), and the proteasomal degradation of endogenous Lnp was slow (Fig. 6). The proteasome inhibitors partially restored the protein level of Lnp in the CAND1 knocked-down cells (Fig. 6b), implying that another mechanism is responsible for the turnover of ubiquitinated Lnp. Recent studies have shown that Lnp is required for the Lst1/SEC24C-mediated ER-phagy^47,48^. Therefore, ubiquitinated Lnp might be removed by ER-phagy in addition to the ubiquitin-proteasome system.

Overall, our results underscore that CAND1 is a positive regulator of Lnp, concomitantly implying the inverse association between the oligomerization and the ubiquitin ligase activity of Lnp for stabilization of the three-way junctions.

## Materials and Methods

### Plasmids

A cDNA encoding the human Lnp was amplified from human cDNA library and subcloned into the pGEX-6P-1 vector (GE Healthcare), the pET28a(+) vector (Novagen), the pCA vector with the C-terminal FLAG tag (pCA-FLAG), and the pCMV vector with the C-terminal mCherry (Thermo Fisher Scientific, pCMV-mCherry). The Lnp mutant with all the lysine residues substituted with arginine (K0) was synthesized by Eurofins Genomics. Lnp ΔTM was generated by substituting the transmembrane domains of Lnp (45–100 aa) with a polypeptide linker (GGS)_3_ by site-directed mutagenesis. The Lnp mutants (K11R, K23R, K34R, K40R, K100R, and K0/R100K) were generated by site-directed mutagenesis. The Lnp mutant comprising the N-terminal cytoplasmic domain of Lnp (1–45 aa, Ub) was subcloned to the pGEX-6P-1 vector. Lnp ΔTM, the Lnp mutants lacking Lnp Ub (ΔUb), the coiled-coil domain (100–143 aa, ΔCC), and the zinc finger motif and the following LNPARK amino acids (275–309 aa, ΔZn + LNPARK) were subcloned to the pET28a(+) vector. The Lnp mutant containing the N-terminal 100 amino acids (N100), K11R, K23R, K34R, K40R, K100R, K0, and K0/R100K were subcloned into the pCA-FLAG vector. Lnp K100R was subcloned into the pCMV-mCherry vector. A cDNA encoding human CAND1 (ID: FHC01125) was purchased from the Kazusa DNA Research Institute and subcloned into the pCMV vector with the N-terminal hemagglutinin (HA) tags and the pMAL-C2 vector (New England BioLabs). A cDNA encoding the mouse Rtn3c was amplified from mouse cDNA library and subcloned into the pGEX-6P-1 vector. cDNAs encoding human UBE2D1 (ID: FHC08258) and human gp78 (ID: FHC25706) were purchased from Kazusa DNA Research Institute and subcloned into the pET28a(+) vector and the pCMV vector with the N-terminal myc tag, respectively. The pCMV vector to express HA-ubiquitin was a generous gift from I. Shoji (Kobe University).

### Antibodies

A mouse anti-actin mAb (AC-74, Cat. No. A2228), a mouse anti-myc mAb (9E10, Cat. No. M5546), a rabbit anti-FLAG pAb (Cat. No. F7425), and a rabbit anti-HA pAb (Cat. No. H6908) were purchased from Sigma-Aldrich. A mouse anti-CLIMP-63 mAb (G1/296, Cat. No. ALX-804-604) was purchased from Enzo Life Sciences. A mouse anti-HA mAb (16B12, Cat. No. MMS-101R) was purchased from COVANCE. A rabbit anti-lunapark pAb (Cat. No. ABS1624) was purchased from EMD Millipore. A mouse anti-TIP120A/CAND1 mAb (G-3, Cat. No. sc-137055) was purchased from Santa Cruz Biotechnology. A rabbit anti-mCherry pAb (Cat. No. ab183628) was purchased from Abcam. A mouse mAb IgG1 Isotype Control (G3A1, Cat. No. 5415) was purchased from Cell Signaling.

### Recombinant proteins

MBP-CAND1 and MBP alone were expressed in *Escherichia coli* (*E*. *coli*) Rosetta-Gami harbouring pMAL-C2-CAND1 and pMAL-C2, respectively, and extracted with buffer A [20 mM Tris-HCl (pH 7.5), 150 mM NaCl, 0.1 mM ZnSO_4_, 1 mM DTT] supplemented with 10 μM APMSF, 10 μg/ml leupeptin, and 5 μg/ml aprotinin, followed by affinity purification with amylose resin (New England BioLabs). MBP-CAND1 and MBP alone were eluted with buffer A supplemented with 20 mM maltose.

GST-Lnp, GST-Lnp Ub, GST-Rtn3c, and GST alone were expressed in *E*. *coli* BL21 (DE3) harbouring pGEX6P-1-Lnp, pGEX6P-1-Lnp Ub, pGEX6P-1-Rtn3c, and pGEX6P-1, respectively, and extracted with buffer A containing 1% Triton X-100, supplemented with 10 μM APMSF, 10 μg/ml leupeptin, and 5 μg/ml aprotinin, followed by affinity purification with glutathione Sepharose (GE Healthcare). GST-Lnp Ub and GST alone were eluted with buffer A containing 1% Triton X-100, supplemented with 10 mM glutathione.

His-Lnp, His-Lnp ΔUb, His-Lnp ΔTM, His-Lnp ΔCC, His-Lnp ΔZn + LNPARK, and His-UBE2D1 were expressed in *E*. *coli* BL21 (DE3) harbouring pET28a(+)-Lnp, pET28a(+)-Lnp ΔUb, pET28a(+)-Lnp ΔTM, pET28a(+)-Lnp ΔCC, pET28a(+)-Lnp ΔZn + LNPARK, and pET28a(+)-UBE2D1, respectively, and extracted with buffer B [50 mM Tris-HCl (pH 8.0), 300 mM NaCl, and 1% Triton X-100] containing 10 mM imidazole, supplemented with 10 μM APMSF, 10 μg/ml leupeptin, and 5 μg/ml aprotinin, followed by affinity purification with Ni-agarose (QIAGEN). His-Lnp and His-UBE2D1 were eluted with buffer B containing 500 mM imidazole. His-E1 protein was a generous gift from I. Shoji (Kobe University).

Lnp-FLAG was expressed in HEK293 cells by the transfection of pCA-Lnp-FLAG. The cells were lysed with buffer C [50 mM HEPES-NaOH, 120 mM NaCl, 2 mM MgCl_2_, and 1% NP-40] supplemented with 10 μM APMSF, 10 μg/ml leupeptin, and 5 μg/ml aprotinin and pre-absorbed with Protein G Fast Flow. The supernatants were incubated with the anti-FLAG M2 affinity gel (Sigma-Aldrich) at 4 °C for 3 h. After being extensively washed with buffer C and subsequently with buffer D [25 mM Tris-HCl (pH 7.4), 20 mM NaCl, 0.1 mM DTT, and 0.1% Triton X-100], Lnp-FLAG were eluted by buffer D containing 100 μg/ml 3x FLAG peptide (Sigma).

### Affinity chromatography

GST-Lnp and GST alone were immobilized on glutathione Sepharose. *Sus scrofa* brains were homogenized in buffer A supplemented with 1 mM MgCl_2_, 1 mM CaCl_2_, 100 μM PMSF, 10 μg/ml leupeptin, and 10 μg/ml aprotinin, and extracted with 0.2% Triton X-100 at 4 °C for 1 h. The sample was centrifuged at 4,200 × *g* at 4 °C for 50 min, followed by further centrifugation at 100,000 × *g* at 4 °C for 1 h. The supernatant was pre-absorbed with glutathione Sepharose to remove the native GST and incubated with immobilized GST-Lnp or GST alone at 4 °C overnight. After being extensively washed with buffer A supplemented with 1 mM MgCl_2_ and 1 mM CaCl_2_, the bound proteins were eluted with buffer E [20 mM Tris-HCl (pH 7.5), 2 M NaCl, 5 mM EDTA, 1 mM DTT, and 1% Triton X-100] and subjected to SDS-PAGE followed by silver staining. The bands of interest were cut out from the gel and digested with trypsin, followed by mass spectrometry analysis.

### Co-immunoprecipitation

For the co-immunoprecipitation assay of overexpressed Lnp, CAND1, and gp78, appropriate combinations of pCA-Lnp-FLAG, pCMV-HA-CAND1, and pCMV5-myc-gp78 were transfected into HEK293 cells with polyethylenimine (Polysciences) and the cells were cultured for 48 h. The cells were lysed with buffer F [20 mM Tris-HCl (pH 7.5), 150 mM NaCl, 1 mM MgCl_2_, and 1% NP-40] supplemented with 10 μM APMSF, 10 μg/ml leupeptin, and 5 μg/ml aprotinin. The lysates were pre-absorbed with protein G Fast Flow (GE Healthcare) at 4 °C for 15 min to remove the proteins binding to the resin nonspecifically. The supernatants were incubated with the anti-HA mAb or the anti-myc mAb immobilized on Protein G Fast Flow at 4 °C for 3 h. After being extensively washed with buffer F, the immunoprecipitates were subjected to SDS-PAGE followed by immunoblotting with the anti-FLAG pAb, the anti-HA pAb, and the anti-myc mAb.

For the co-immunoprecipitation assay of endogenous CAND1 and endogenous Lnp, HeLa cells were lysed with buffer F supplemented with 10 μM APMSF, 10 μg/ml leupeptin, and 5 μg/ml aprotinin and pre-absorbed with Protein G Fast Flow in the same manner as described above. The supernatants were incubated with the anti-CAND1 mAb or the mouse control IgG immobilized on Protein G Fast Flow at 4 °C overnight. After being extensively washed with buffer F, the immunoprecipitates were subjected to SDS-PAGE followed by immunoblotting with the anti-CAND1 mAb and the anti-Lnp pAb.

### Binding assay

For the binding assay of Lnp or Rtn3c and CAND1, GST-Lnp (1 nmol) or GST-Rtn3c (1 nmol) immobilized on glutathione Sepharose was incubated with MBP-CAND1 (10 nmol) or MBP alone (10 nmol) in buffer G [50 mM Tris-HCl (pH 8.0), 150 mM NaCl, 0.1 mM ZnSO_4_, 1 mM MgCl_2_, 1 mM CaCl_2_, and 1% Triton X-100] containing 10 mM imidazole at 4 °C for 2 h. After being extensively washed with buffer G containing 10 mM imidazole, the bound proteins were subjected to SDS-PAGE followed by CBB staining. For the reciprocal binding assay, MBP-CAND1 (0.6 nmol) immobilized on amylose resin was incubated with GST-Lnp or GST-Rtn3c (3 nmol) in buffer A containing 1% Triton X-100 at 4 °C for 2 h. After being extensively washed with buffer A containing 1% Triton X-100, the bound proteins were subjected to SDS-PAGE followed by CBB staining.

For the binding assay of the deletion mutants of Lnp (Lnp ΔUb, ΔTM, ΔCC, and ΔZn + LNPARK) and CAND1, His-Lnp WT (0.5 nmol), ΔUb (0.5 nmol), ΔTM (0.5 nmol), ΔCC (0.5 nmol), or ΔZn + LNPARK (0.5 nmol) immobilized on Ni-agarose was incubated with MBP-CAND1 (3 nmol) or MBP alone (3 nmol) in buffer G containing 10 mM imidazole at 4 °C for 2 h. After being extensively washed with buffer G containing 10 mM imidazole, the bound proteins were subjected to SDS-PAGE followed by CBB staining.

For the binding assay of Lnp ΔUb and Lnp Ub, His-Lnp ΔUb (0.6 nmol) immobilized on Ni-agarose was incubated with GST-Lnp Ub (3 nmol) or GST alone (3 nmol) in buffer G containing 20 mM imidazole and 1 mM DTT at 4 °C for 2 h. After being extensively washed with buffer G containing 20 mM imidazole and 1 mM DTT, the bound proteins were subjected to SDS-PAGE followed by CBB staining. To examine the effect of CAND1 on the binding of Lnp ΔUb and Lnp Ub, His-Lnp ΔUb (0.6 nmol) immobilized on Ni-agarose was incubated with GST-Lnp Ub (1.2 nmol) and MBP-CAND1 (6 nmol) or MBP alone (6 nmol) in buffer G containing 20 mM imidazole and 1 mM DTT at 4 °C for 2 h. After being extensively washed with buffer G containing 20 mM imidazole and 1 mM DTT, the bound proteins were subjected to SDS-PAGE followed by CBB staining.

For the binding assay of Lnp Ub and CAND1, GST-Lnp (0.6 nmol) or GST-Lnp Ub (0.6 nmol) immobilized on glutathione Sepharose was incubated with MBP-CAND1 (6 nmol) in buffer A containing 1% Triton X-100 at 4 °C for 2 h. After being extensively washed with buffer A containing 1% Triton X-100, the bound proteins were subjected to SDS-PAGE followed by CBB staining.

### Sucrose density gradient ultracentrifugation

His-Lnp (0.6 nmol) was incubated with MBP-CAND1 (2 nmol) in buffer H [20 mM Tris-HCl (pH 7.5), 150 mM NaCl, and 1% Triton X-100] at 4 °C for 1 h. The mixture was loaded on top of a 10–40% (w/v) linear sucrose gradient in buffer H and subjected to ultracentrifugation at 200,000 × *g* at 4 °C for 20 h. The gradient was fractionated into seventeen fractions (300 μl per fraction) from the bottom to the top. The fractions were subjected to SDS-PAGE followed by CBB staining. To estimate the molecular weights of the fractionated samples, Gel Filtration Standard (Bio-Rad, Cat. No. 151–1901) was used on a parallel gradient. The intensities of the bands corresponding to His-Lnp were quantified by ImageJ. For pull-down assay, the odd-numbered fractions of the mixture of His-Lnp and MBP-CAND1 were diluted with 12 volumes of buffer B containing 10 mM imidazole, and incubated with Ni-agarose at 4 °C for 2 h. After being extensively washed with buffer B containing 10 mM imidazole, the bound proteins were subjected to SDS-PAGE followed by CBB staining.

### Ubiquitination assay

For *in vitro* ubiquitination assay using Lnp-FLAG, Lnp-FLAG (0.7 μM) was incubated with His-E1 (80 nM), His-UBE2D1 (4 μM), 2 mM magnesium/ATP, and MBP-CAND1 (0.35 or 0.7 μM) or MBP alone (0.35 or 0.7 μM) in buffer D at room temperature for 30 min. HA-ubiquitin (30 μM, Boston Biochem, Cat. No. U-110) was added to the samples and incubated at 37 °C for 150 min. The samples were subjected to SDS-PAGE followed by immunoblotting with the anti-HA mAb and the anti-FLAG pAb.

For *in vitro* ubiquitination assay using GST-Lnp Ub, GST-Lnp Ub (2 μM) was incubated with His-E1 (640 nM), His-UBE2D1 (4 μM), 2 mM magnesium/ATP, and MBP-CAND1 (1.4 μM) or MBP alone (1.4 μM) in buffer D at room temperature for 30 min. HA-ubiquitin (30 μM) was added to the samples and incubated at 37 °C for 120 min. The samples were subjected to SDS-PAGE followed by immunoblotting with the anti-HA mAb.

For *in vivo* ubiquitination assay, appropriate combinations of pCMV-HA-ubiquitin, pCMV-HA-CAND1, and pCA-Lnp-FLAG were transfected into HEK293 cells and the cells were cultured for 48 h. After the cells were incubated with 20 μM MG132 (Nacalai tesque, Cat. No. 09494-22) for 4 h, they were lysed with buffer C supplemented with 10 μM APMSF, 10 μg/ml leupeptin, and 5 μg/ml aprotinin and pre-absorbed with Protein G Fast Flow. The supernatants were heated at 90 °C for 5 min in the presence of 1% SDS to allow Lnp-FLAG to denature and dissociate from the binding proteins. The samples were diluted with ten volumes of buffer C for renaturation and incubated with anti-FLAG M2 affinity gel at 4 °C for 3 h. After being extensively washed with buffer C, the immunoprecipitates were subjected to SDS-PAGE followed by immunoblotting with the anti-HA pAb and the anti-FLAG pAb. To check the expression of HA-CAND1, the input samples were subjected to SDS-PAGE followed by immunoblotting with the anti-CAND1 mAb.

### siRNA-mediated knockdown

Silencer Select siRNAs, siRNA ID: s31616 (siCAND1 #1), siRNA ID: s31617 (siCAND1 #2), siRNA ID: s31618 (siCAND1 #3), siRNA ID: s228373 (siCul1), siRNA ID: s12889 (siSkp1), and Silencer Select Negative Control No. 2 siRNA (Cat. No. 4390846) were purchased from Thermo Fisher Scientific.

For knocking down CAND1, 10 nM of siCAND1 #1–3 or the control siRNA was transfected into COS-7 cells using Lipofectamine RNAiMAX (Thermo Fisher Scientific) and the cells were cultured for 96 h. The total cell extracts of the cells were subjected to SDS-PAGE followed by immunoblotting with the anti-CAND1 mAb, the anti-Lnp pAb, and the anti-actin mAb. The intensities of the immunoreactive bands for Lnp and actin were quantified by ImageJ. To inhibit the proteasome degradation of Lnp in the CAND1 knocked-down cells by clasto-lactacystin β-lactone (cLbetaL, Abcam, Cat. No. ab141412), siCAND1 #2 or the control siRNA was transfected into COS-7 cells and the cells were cultured for 72 h. 10 μM cLbetaL or water was added to the medium and the cells were cultured for 24 h. To inhibit the proteasome degradation of Lnp in the CAND1 knocked-down cells by MG132, siCAND1 #2 or the control siRNA was transfected into COS-7 cells and the cells were cultured for 88 h. 20 μM MG132 or DMSO was added to the medium and the cells were cultured for 8 h.

To examine the resistancy of Lnp K100R to the effects of CAND1 knockdown, pCMV-Lnp-mCherry or pCMV-Lnp K100R-mCherry was co-transfected with siCAND1 #2 into COS-7 cells with Lipofectamine 2000 (Thermo Fisher Scientific). The cells were split into three dishes and cultured for 24, 48, or 72 h, respectively. The total cell lysates were subjected to SDS-PAGE followed by immunoblotting with the anti-mCherry pAb, the anti-CAND1 mAb, and the anti-actin mAb.

For knocking down Cul1 or Skp1, 10 nM of siCul1, siSkp1, or the control siRNA was transfected into COS-7 cells and the cells were cultured for 96 h. Total RNA was extracted from COS-7 cells with RNeasy Mini kit (QIAGEN). 0.6 μg of total RNA was used for reverse transcription by Superscript Vilo (Thermo Fisher Scientific). Quantitative PCR was performed on LightCycler^TM^ 480 Real Time PCR System (Roche) using KAPA SYBR FAST Master Mix (KAPA BIOSYSTEMS). GAPDH was used as an internal control. PCR experiments were performed in duplicate, and standard deviations were calculated and displayed as error bars.

### Immunostaining

For observing the localization of Lnp and CAND1, pCMV-Lnp-mCherry was transfected into COS-7 cells and the cells were cultured for 24 h. The cells were fixed with 4% paraformaldehyde and permeabilized with 0.2% Triton X-100 in PBS. After blocking with PBS supplemented with 1% BSA, the cells were incubated with the anti-CAND1 mAb, followed by incubation with the secondary antibody conjugated with Alexa Flour^TM^ 488 (Thermo Fisher Scientific, Cat. No. A11029).

To visualize the entire ER structures, the baculovirus carrying ER-RFP (CellLight™ ER-RFP BacMam 2.0, Thermo Fisher Scientific, Cat. No. C10591) was transfected into the cells and the cells were cultured for 16 h.

To immunostain the cells for CLIMP-63, the cells were fixed with 4% paraformaldehyde and permeabilized with 0.2% Triton X-100 in PBS. After blocking with PBS supplemented with 1% BSA, the cells were incubated with the anti-CLIMP-63 mAb, followed by incubation with the secondary antibody conjugated with Alexa Flour^TM^ 488. To express Lnp-mCherry, Lnp K100R-mCherry, or mCherry alone in the CAND1 knocked-down cells, siCAND1 #2 was transfected into COS-7 cells and the cells were cultured for 72 h. pCMV-Lnp-mCherry, pCMV-Lnp K100R-mCherry, or pCMV-mCherry was transfected into the cells with Lipofectamine 2000 and the cells were cultured for 24 h.

The cells were embedded and viewed using a confocal laser scanning microscope (Carl Zeiss, LSM 510 META) using a 63× oil-immersion objective lens. Collected data were exported as 8-bit TIFF files and processed using Adobe Photoshop. The intensities of ER-RFP were plotted by ImageJ. The CLIMP-63-positive area and the total cell area were quantified by ImageJ.

## Supplementary information

Supplementary information
