# Supplementary material for: CAND1 regulates lunapark for the proper tubular network of the endoplasmic reticulum
Source: Sci Rep. 2019 Sep 11;9:13152. doi: 10.1038/s41598-019-49542-x (PMC6739345; doi:10.1038/s41598-019-49542-x)
Supplement: Supplementary file 1 — Supplementary information [file 41598_2019_49542_MOESM1_ESM.pdf]

Supplementary information

CAND1 regulates lunapark for the proper tubular network of the endoplasmic reticulum

Hiroaki Kajiho, Yasunori Yamamoto, Toshiaki Sakisaka*
Division of Membrane Dynamics, Department of Physiology and Cell Biology, Kobe University Graduate School of Medicine, Kobe, 650-0017, Japan.

*Address correspondence to Toshiaki Sakisaka, Division of Membrane Dynamics, Department of Physiology and Cell Biology, Kobe University Graduate School of Medicine, Kobe 650-0017, Japan
Tel.: +81-78-382-5727
Fax: +81-78-382-5419
E-mail: sakisaka@med.kobe-u.ac.jp

Supplementary figure legends

Supplementary table S1. The list of all the proteins identified from the three bands (p130, p110, and p102) in Figure 1a.

The scores indicate their Mascot scores.

Supplementary figure S1. Lunapark directly binds to CAND1.

MBP-CAND1 immobilized on amylose resin was incubated with GST-Lnp or GST-Rtn3c. After being extensively washed, the bound proteins were subjected to SDS-PAGE followed by CBB staining.

Supplementary figure S2. The ubiquitin ligase domain, the transmembrane domain, and the coiled-coiled domain of lunapark are important for the binding to CAND1.

(a) Schematic representation of the primary structure of Lnp and the deletion mutants used in this study. (b) Reduction of the direct binding of Lnp to CAND1 by deleting Ub, TM, or CC. WT or the deletion mutants of His-Lnp immobilized on Ni-agarose were incubated with MBP alone or MBP-CAND1. After being extensively washed, the bound proteins were subjected to SDS-PAGE followed by CBB staining.

Supplementary figure S3. Lunapark and CAND1 do not co-localize at the three-way junctions.

COS-7 cells were transfected with Lnp-mChe. The cells were immunostained with the anti-CAND1 mAb. The yellow dashed line indicates the edge of the cell. Scale bar, 10 μm.

Supplementary figure S4. The lunapark-CAND1 and the lunapark-gp78 complex are formed individually.

(a) No binding of CAND1 to the Lnp-gp78 complex. HEK293 cells were transfected with the indicated combinations of HA-CAND1, Lnp-FLAG, and gp78-myc. NP-40 extracts of the transfected cells were immunoprecipitated with the anti-myc mAb, followed by immunoblotting with the anti-HA pAb, the anti-FLAG pAb, and the anti-myc mAb. (b) No binding of gp78 to the Lnp-CAND1 complex. HEK293 cells were transfected with the indicated combinations of gp78-myc, Lnp-FLAG, and HA-CAND1. NP-40 extracts

of the transfected cells were immunoprecipitated with the anti-HA pAb, followed by immunoblotting with the anti-myc mAb, the anti-FLAG pAb, and the anti-HA pAb.

Supplementary figure S5. CAND1 does not affect the ubiquitin ligase activity of the N-terminal region of lunapark.

(a) No direct binding of CAND1 to the N-terminal cytoplasmic domain of Lnp. GST-Lnp Ub or GST WT-Lnp immobilized on glutathione Sepharose was incubated with MBP-CAND1. After being extensively washed, the bound proteins were subjected to SDS-PAGE followed by CBB staining. (b) No reduction of the ubiquitination activity of the N-terminal cytoplasmic domain of Lnp by CAND1. Indicated combinations of GST-Lnp Ub, MBP alone, or MBP-CAND1 were incubated with His-E1, His-UBE2D1, HA-Ub, and magnesium/ATP at 37°C for 120 min. The samples were subjected to SDS-PAGE followed by immunoblotting with the anti-HA mAb. The arrowheads denote ubiquitin chains. The asterisk denotes the binding of His-E1 bound to HA-Ub.

Supplementary figure S6. The N-terminal 100 amino acids of lunapark is auto-ubiquitinated.

(a) Schematic representation of the primary structure of the Lnp mutant encoding N-terminal 100 amino acids (Lnp N100). (b) Auto-ubiquitination of Lnp N100. HEK293 cells were transfected with empty vector (EV) or Lnp N100-FLAG along with HA-Ub. Lnp N100-FLAG was immunoprecipitated with the anti-FLAG mAb as in Figure 4b, and subjected to SDS-PAGE followed by immunoblotting with the anti-HA pAb and the anti-FLAG pAb. Arrowheads denote the high-molecular-weight Lnp N100-ubiquitin conjugates formed by auto-ubiquitination of Lnp N100-FLAG.

Supplementary figure S7. MG132 moderately restores the protein level of endogenous lunapark in the CAND knocked-down cells.

The COS-7 cells transfected with the control siRNA or siCAND1 #2 were cultured in the presence (+) or absence (-) of MG132 for 8 h. The total cell extracts were subjected to SDS-PAGE followed by immunoblotting with the anti-CAND1 mAb, the anti-Lnp pAb, and the anti-actin mAb. The intensities of immunoreactive bands for Lnp were normalized to those for actin, and the relative band intensities are shown at the bottom.

Supplementary figure S8. The lunapark K100R mutant is more resistant to the effects of CAND1 knockdown than wild type.

Lnp-mChe or Lnp K100R-mChe was co-transfected with siCAND1 #2 into COS-7 cells. The cells were split into three dishes and cultured for 24, 48, or 72 h, respectively. The total cell extracts were subjected to SDS-PAGE followed by immunoblotting with the anti-mCherry pAb, the anti-CAND1 mAb, and the anti-actin mAb. The intensities of immunoreactive bands for Lnp-mChe and Lnp K100R-mChe were normalized to those for actin, and expressed as percentages of Lnp-mChe and Lnp K100R-mChe at 24 h after transfection, respectively. The relative band intensities are shown at the bottom.

Supplementary figure S9. Silencing Cul1 or Skp1 does not alter the morphology of the tubular ER network.

(a) Efficient reductions of the Cul1 and Skp1 mRNA by siRNA transfection. COS-7 cells were transfected with the control siRNA, siRNA targeting Cul1 (siCul1), or siRNA targeting Skp1 (siSkp1). The total RNA was extracted from the cells, and the amount of Cul1 mRNA or Skp1 mRNA in the total RNA was quantified by quantitative RT-PCR. The amounts of Cul1 and Skp1 mRNA were normalized to those of GAPDH mRNA, and expressed as percentages of the control siRNA-transfected cells. Data are the averages ± SEM of three independent experiments. ***$P < 0.001$; paired Student's *t*-test. (b) No obvious expansion of the ER sheets to the peripheral area in the Cul1 or Skp1 knocked-down cells. The COS-7 cells transfected with the control siRNA, siCul1, or siSkp1 were immunostained with the anti-CLIMP-63 mAb. The yellow dashed lines indicate the edges of the cells. Scale bars, 10 μm.

Supplementary figure S10. Restoration of the ER morphology in the CAND1 knocked-down cells by the exogenous expression of the lunapark K100R mutant.

COS-7 cells were transfected with siCAND1 #2 and cultured for 72 h. Then the siCAND1 #2-transfected cells were transfected with mCherry, Lnp-mChe, or Lnp K100R-mChe, and cultured for 24 h. The cells were immunostained with the anti-CLIMP-63 mAb. The yellow dashed lines indicate the edges of the cells. Scale bars, 10 μm.

Supplementary figure S11. Uncropped silver-stained gel for Figure 1a, uncropped

immunoblots for Figures 1c and 1d, and uncropped CBB-stained gel for Figure 1e.
Dashed boxes indicate cropped areas.

Supplementary figure S12. Uncropped CBB-stained gels for Figures 2a, 2b, 3b, and 3c.
Dashed boxes indicate cropped areas.

Supplementary figure S13. Uncropped immunoblots for Figures 4a and 4b.
Dashed boxes indicate cropped areas.

Supplementary figure S14. Uncropped immunoblots for Figures 5a and 5b.
Dashed boxes indicate cropped areas.

Supplementary figure S15. Uncropped immunoblots for Figures 6a and 6b.
Dashed boxes indicate cropped areas.

Supplementary figure S16. Uncropped CBB-stained gels for Supplementary figures S1 and S2b, and uncropped immunoblots for Supplementary figure S4a.
Dashed boxes indicate cropped areas.

Supplementary figure S17. Uncropped immunoblots for Supplementary figure S4b.
Dashed boxes indicate cropped areas.

Supplementary figure S18. Uncropped CBB-stained gel for Supplementary figure S5a and uncropped immunoblot for Supplementary figure S5b.
Dashed boxes indicate cropped areas.

Supplementary figure S19. Uncropped immunoblots for Supplementary figures S6b and S7.
Dashed boxes indicate cropped areas.

Supplementary figure S20. Uncropped immunoblots for Supplementary figure S8.
Dashed boxes indicate cropped areas.

Band	Mass spectrometry identification	Score
p130	Cullin-associated and neddylation-dissociated 1 (CAND1)	1091
	Importin-5	356
	Drebrin	206
	AP-3 complex subunit beta-2	185
	Spectrin alpha chain, non-erythrocytic 1	170
	Exportin-4	169
	Lunapark	142
	Ubiquitin conjugation factor E4 A	116
	Ankyrin-2	101
	Ran-binding protein 6	74
	Sodium/potassium-transporting ATPase subunit alpha-2	72
	Microtubule-associated protein 2	63
	Exportin-1	62
	Rab-like protein 6	58
	Microtubule-associated protein 1B	57
	Importin-7	52
	Eukaryotic translation initiation factor 5B	50
	Serine/threonine-protein kinase mTOR	50
	Elongation factor 1-alpha 1	44
	Polyphosphoinositide phosphatase	40
	Cardiotrophin-like cytokine factor 1	32
	Importin-9	29
	Plasma membrane calcium-transporting ATPase 2	28
	Acyloxyacyl hydrolase	28
	Spectrin beta chain, non-erythrocytic 1	27
	BAI1-associated protein 3	25
p110	Importin-5	1215
	Importin-7	208
	Spectrin alpha chain, non-erythrocytic 1	88
	Drebrin	87
	Lunapark	70
	Importin-11	61
	Exportin-6	53
	Nck-associated protein 1	46
	Alpha-adducin	44
	Exportin-1	39
	Sodium/potassium-transporting ATPase subunit alpha-1	38
	Dynein heavy chain 10, axonemal	30
	AP-2 complex subunit alpha-1	20
	Nesprin-1	16
p102	Exportin-1	1175
	Importin-5	698
	Exportin-2	219
	Microtubule-associated protein 1B	145
	AP-1 complex subunit beta-1	99
	AP-3 complex subunit beta-2	79
	Alpha-actinin-1	65
	Exportin-7	64
	Polyphosphoinositide phosphatase	62
	Lunapark	45
	Beta-adducin	37
	Glycogen phosphorylase, muscle form	35
	Importin-11	32
	Acyloxyacyl hydrolase	28
	Importin-7	28
	Zinc finger C3H1 domain-containing protein	27
	Transportin-3	26
	SRSF protein kinase 2	22

Supplementary table S1

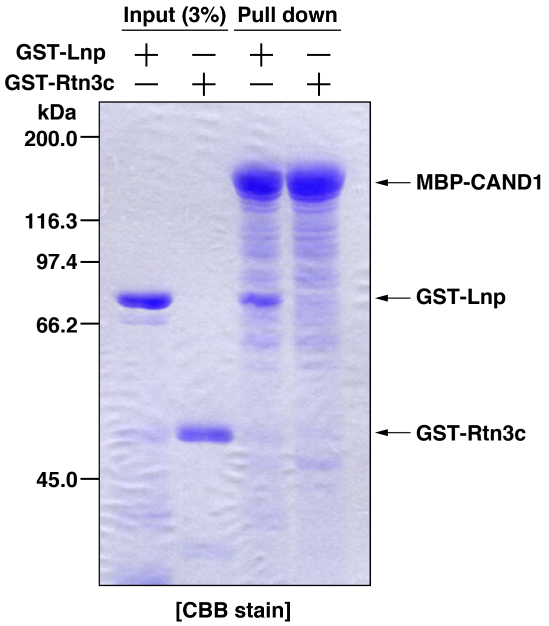

Supplementary figure S1

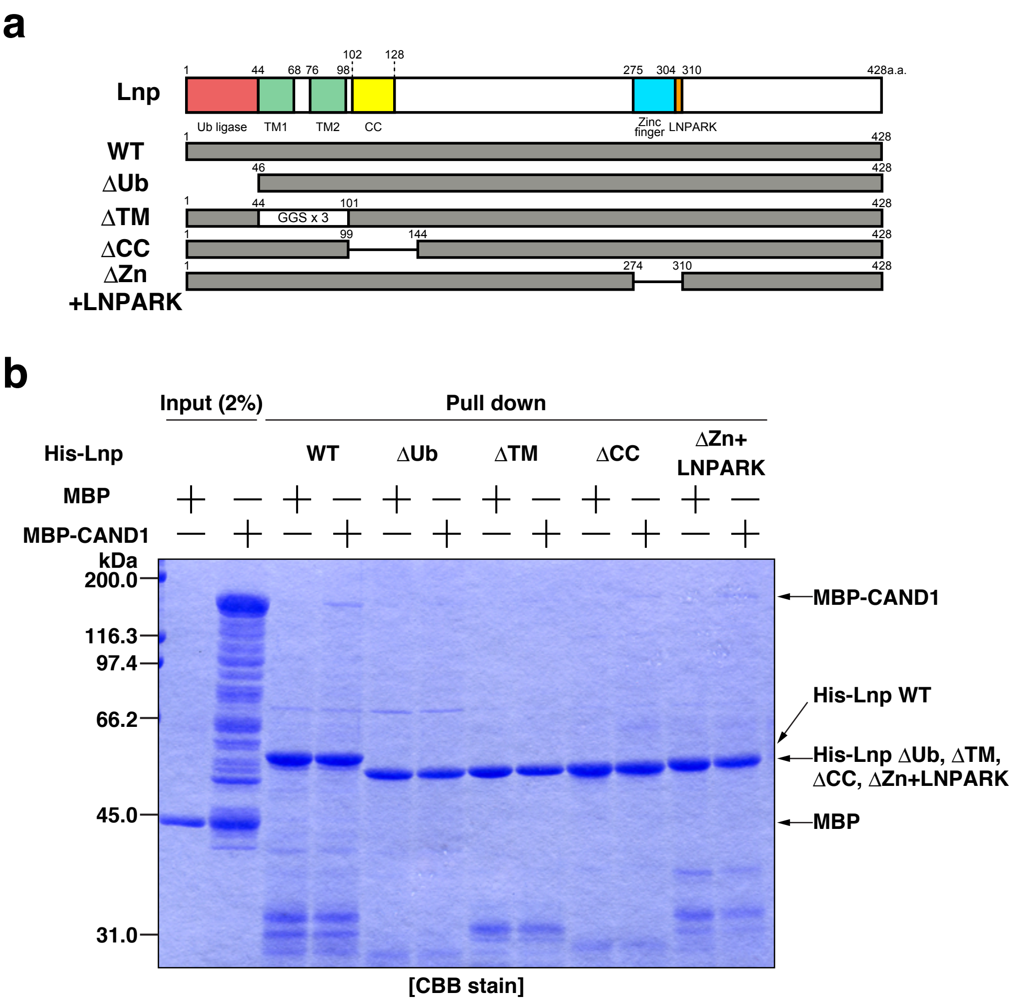

Supplementary figure S2

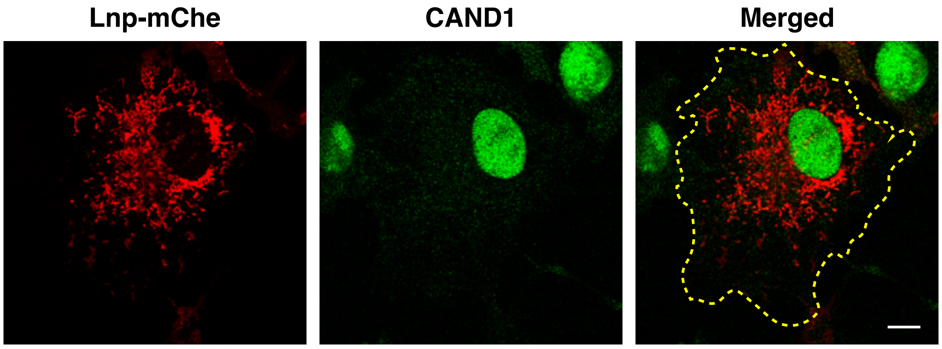

Supplementary figure S3

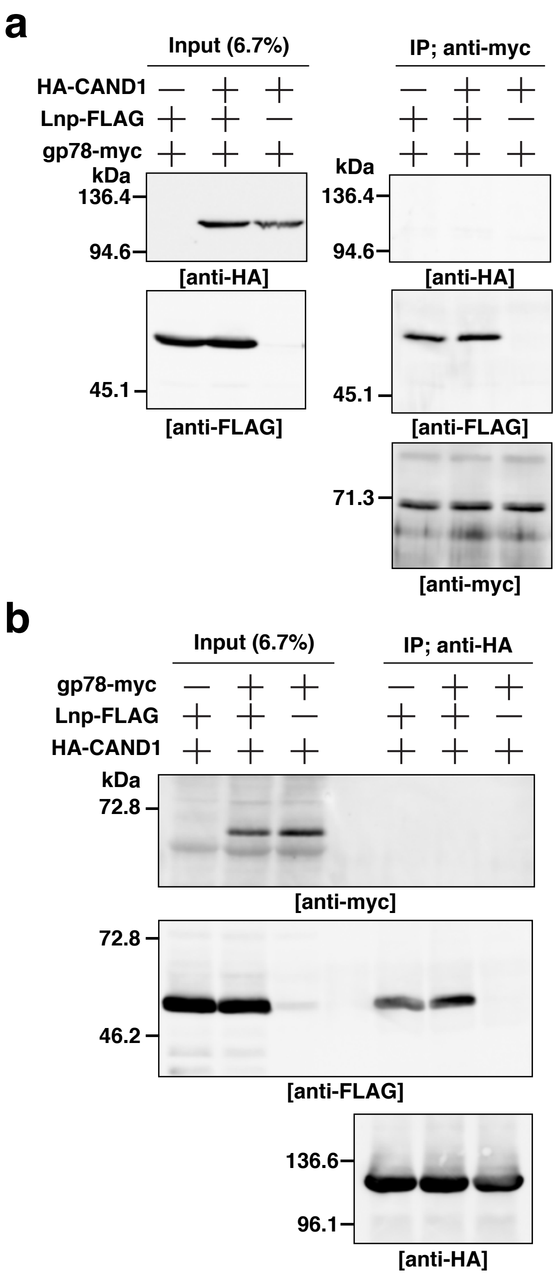

Supplementary figure S4

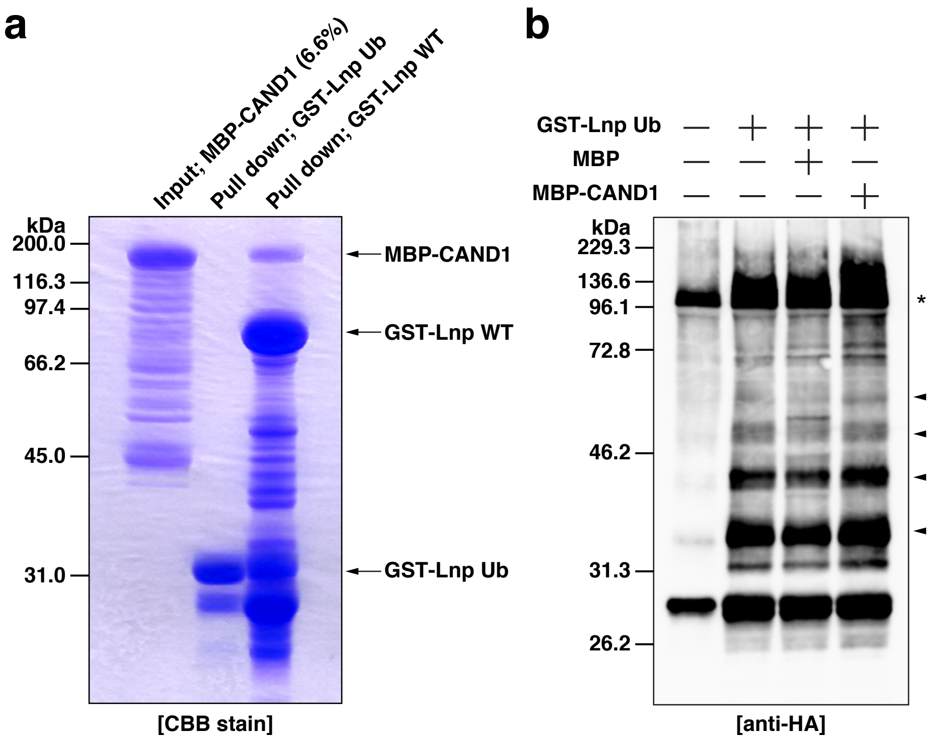

Supplementary figure S5

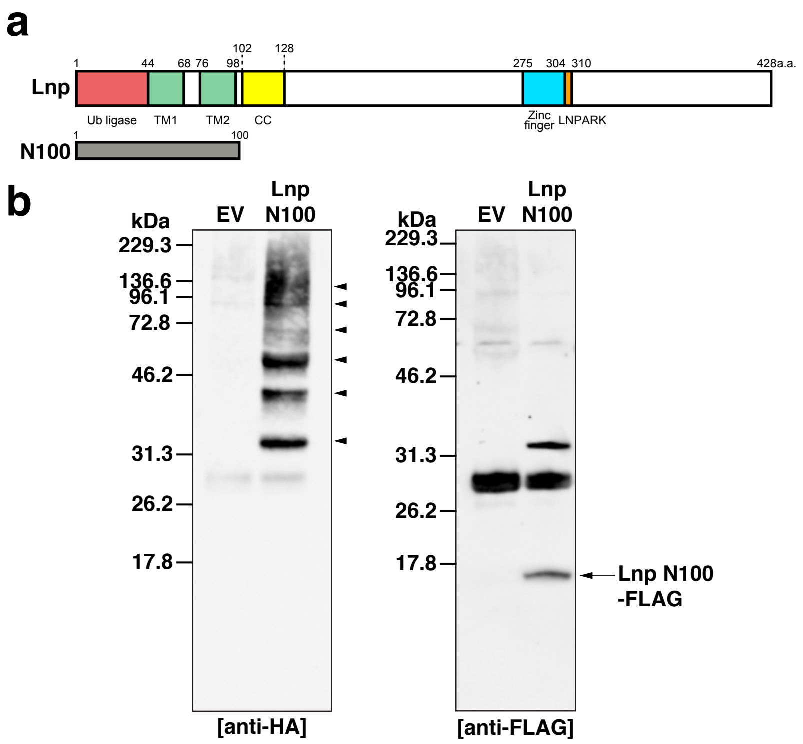

Supplementary figure S6

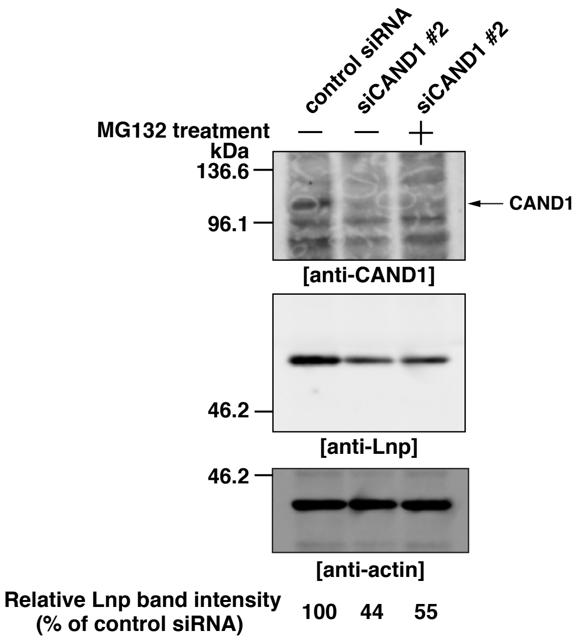

Supplementary figure S7

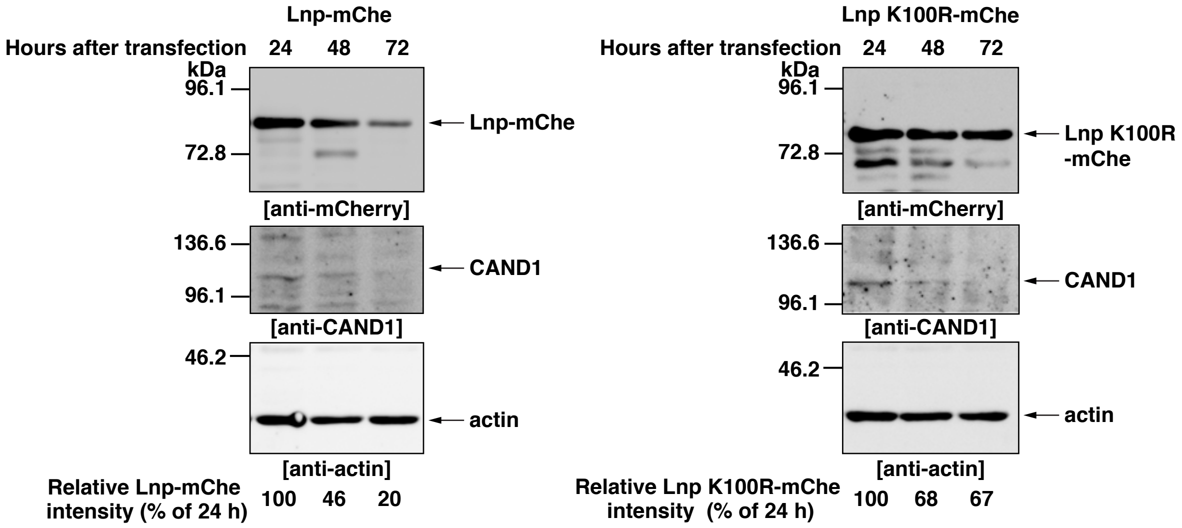

Supplementary figure S8

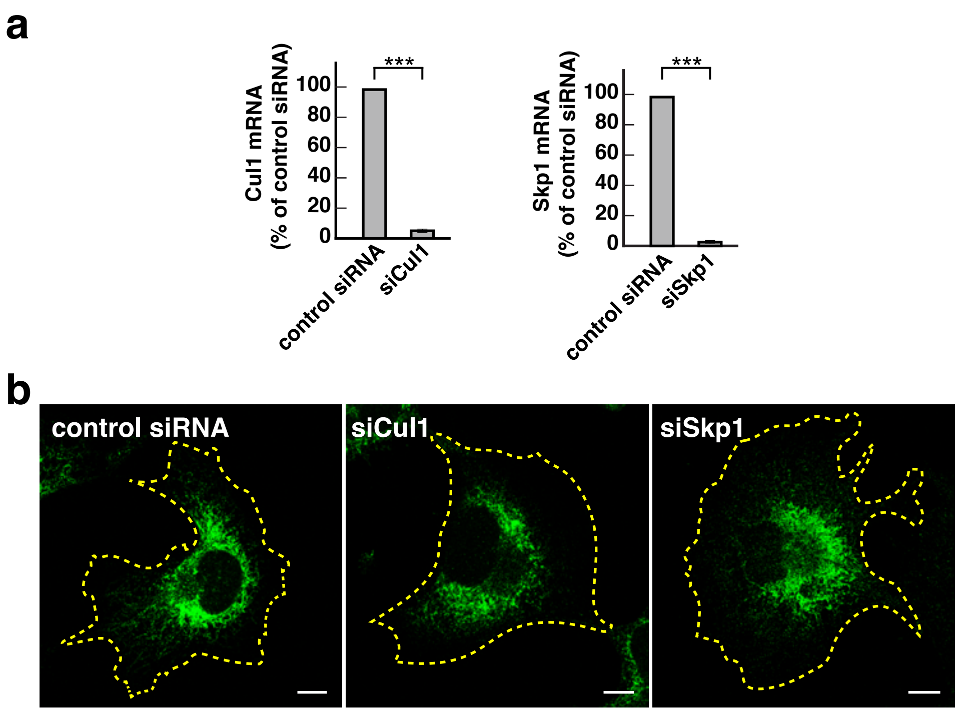

Supplementary figure S9

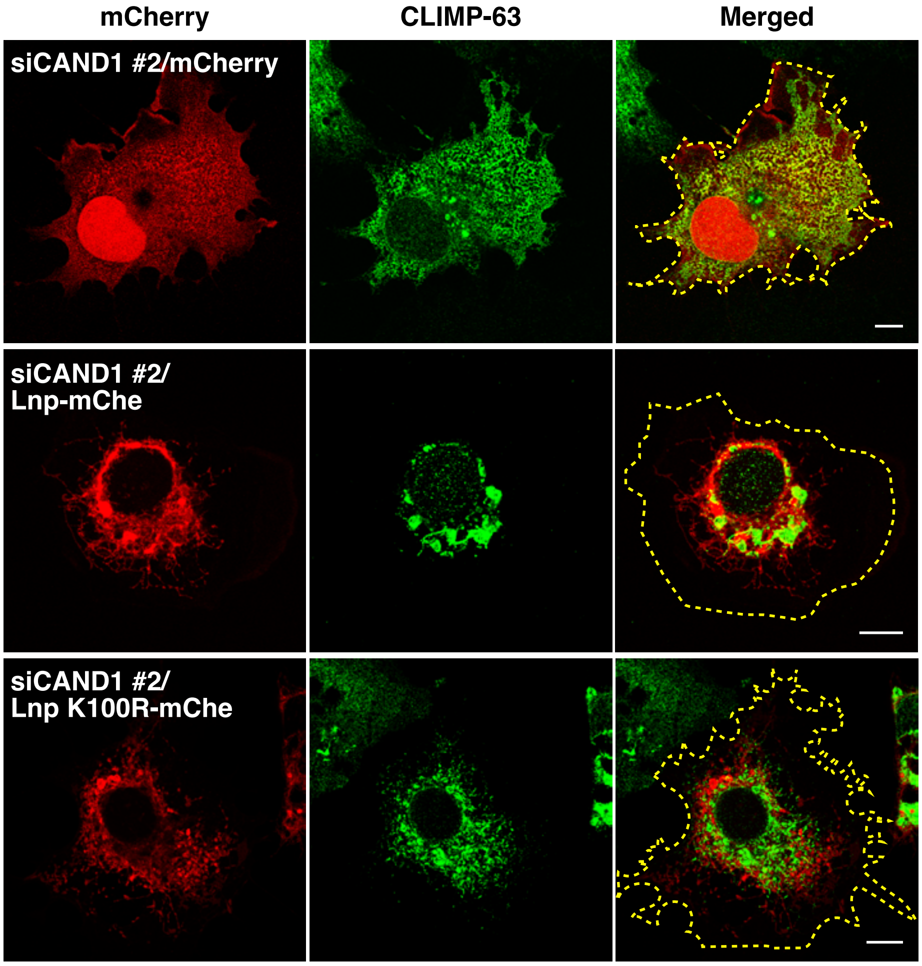

Supplementary figure S10

Figure 1a

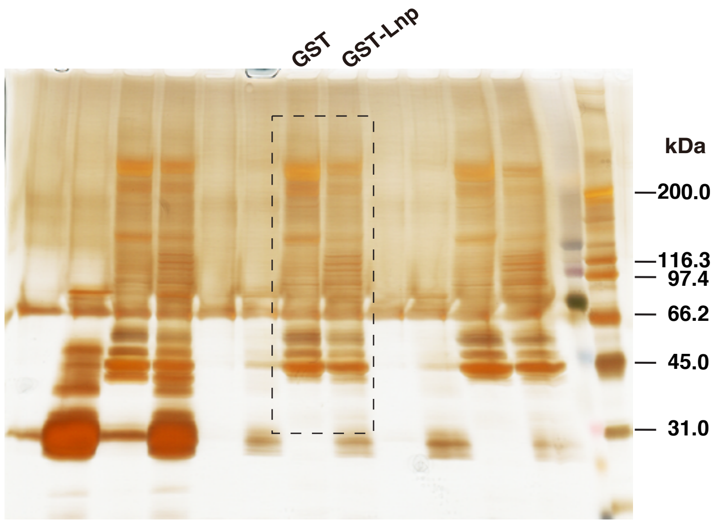

[silver stain]

Figure 1c

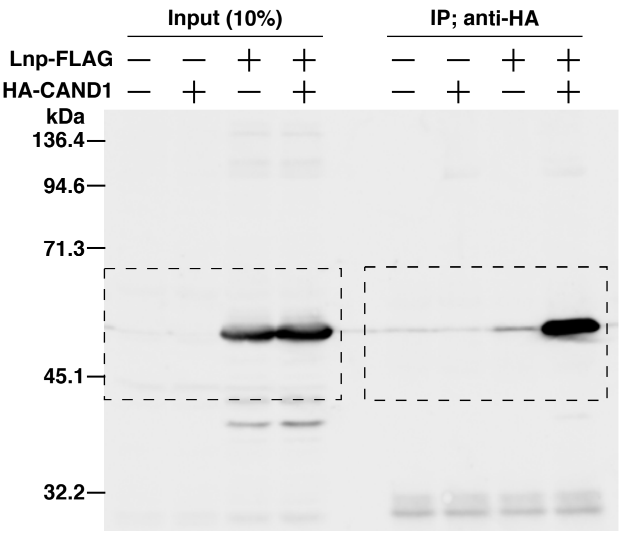

[anti-FLAG]

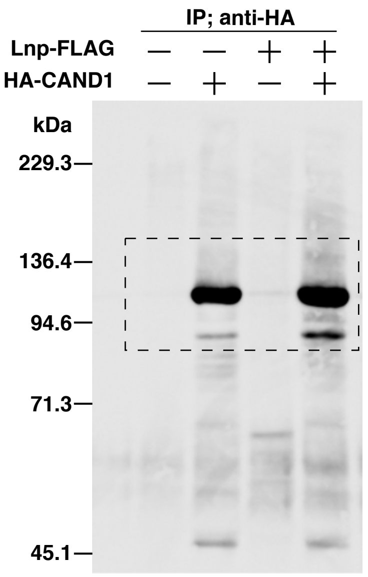

[anti-HA]

Figure 1d

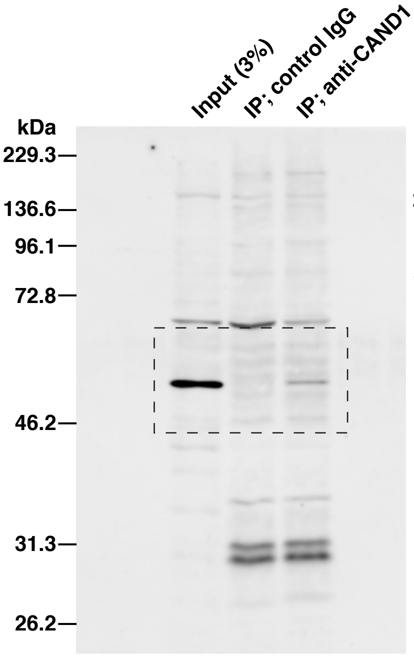

[anti-Lnp]

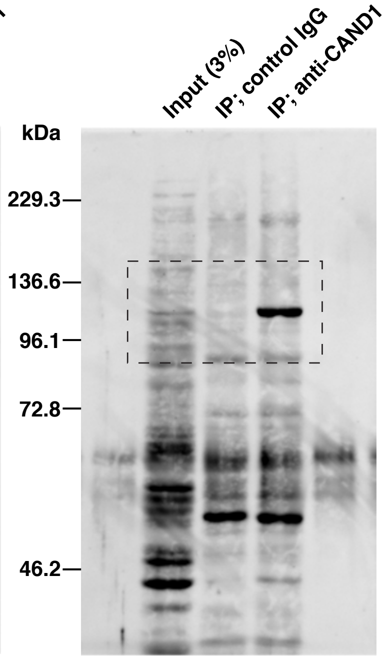

[anti-CAND1]

Figure 1e

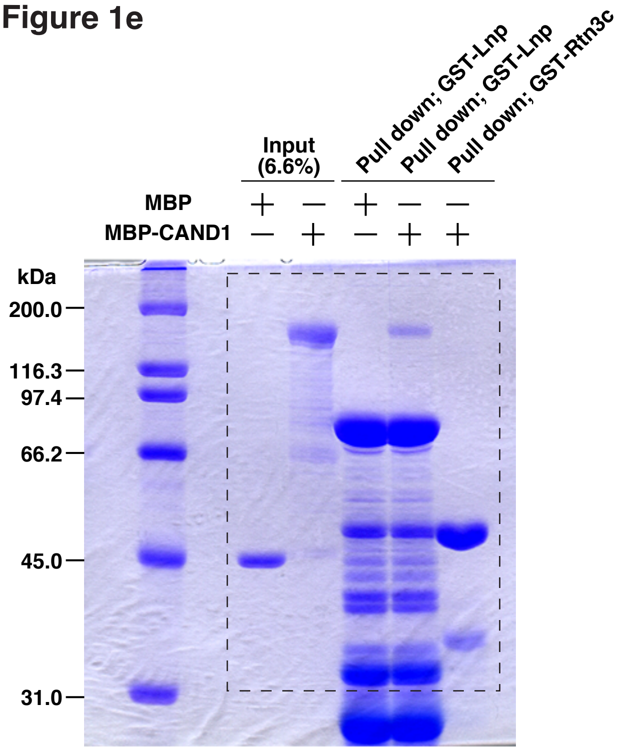

[CBB stain]

Supplementary figure S11

Figure 2a

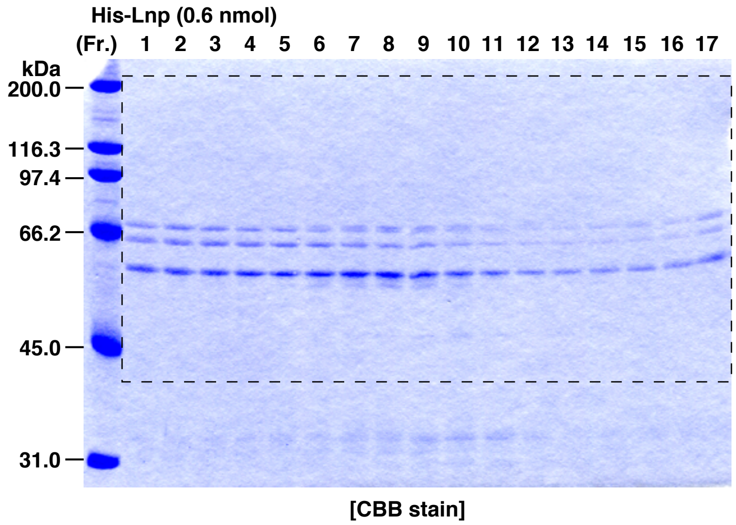

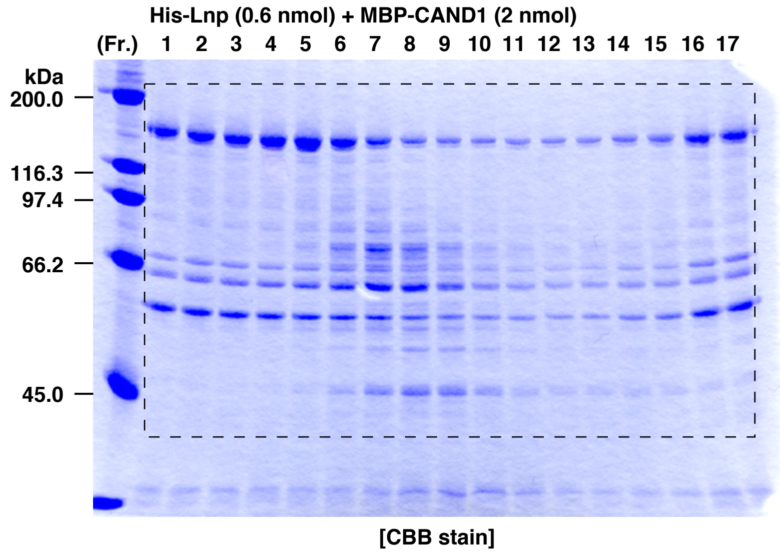

Figure 2b

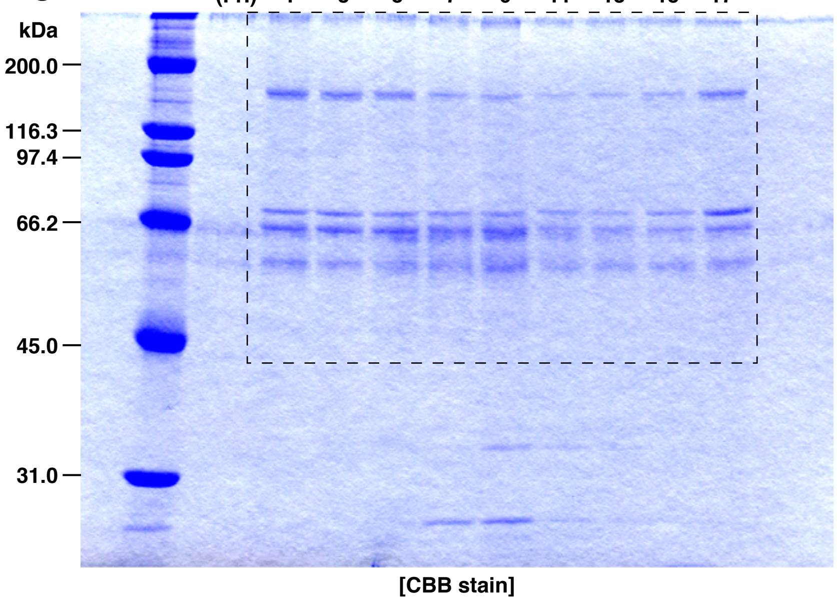

Figure 3b

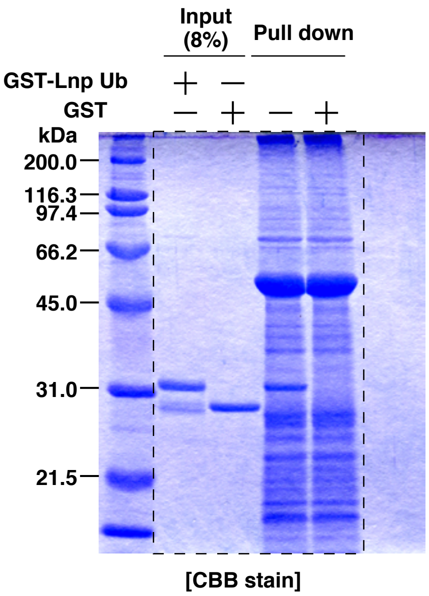

Figure 3c

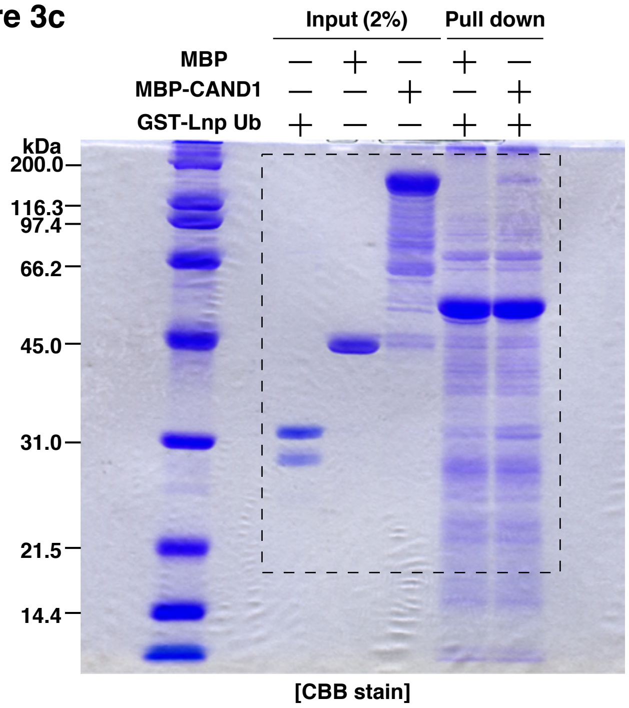

Supplementary figure S12

Figure 4a

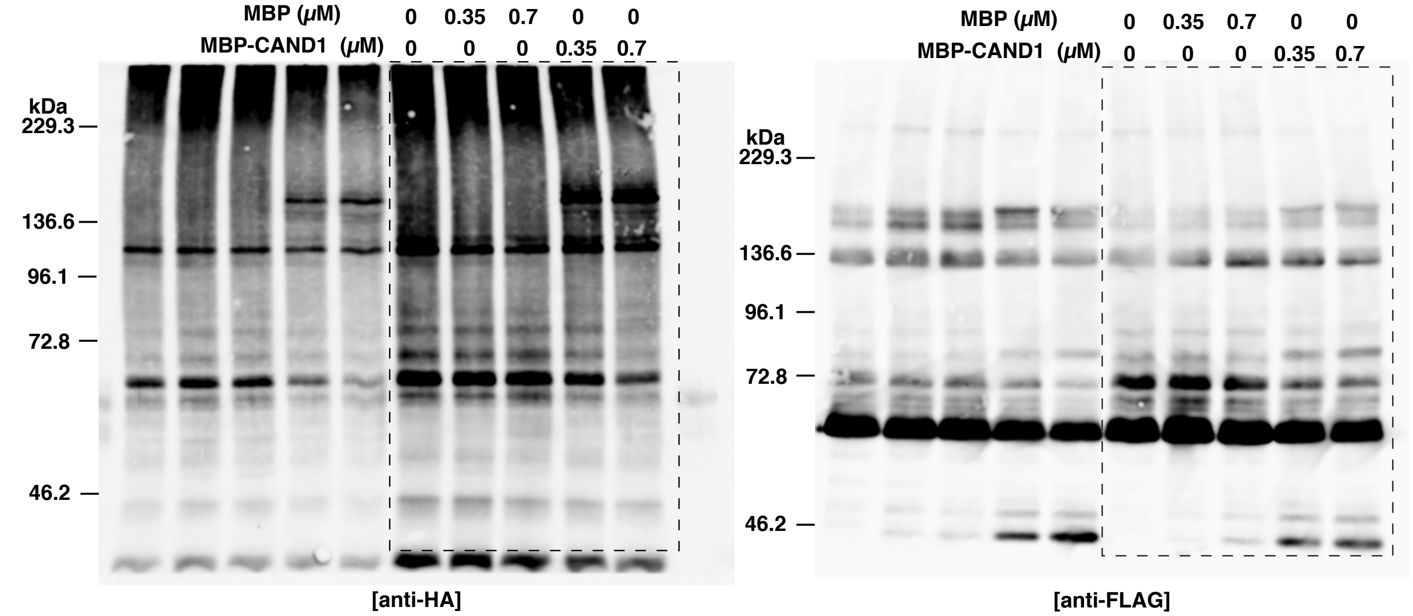

Figure 4b

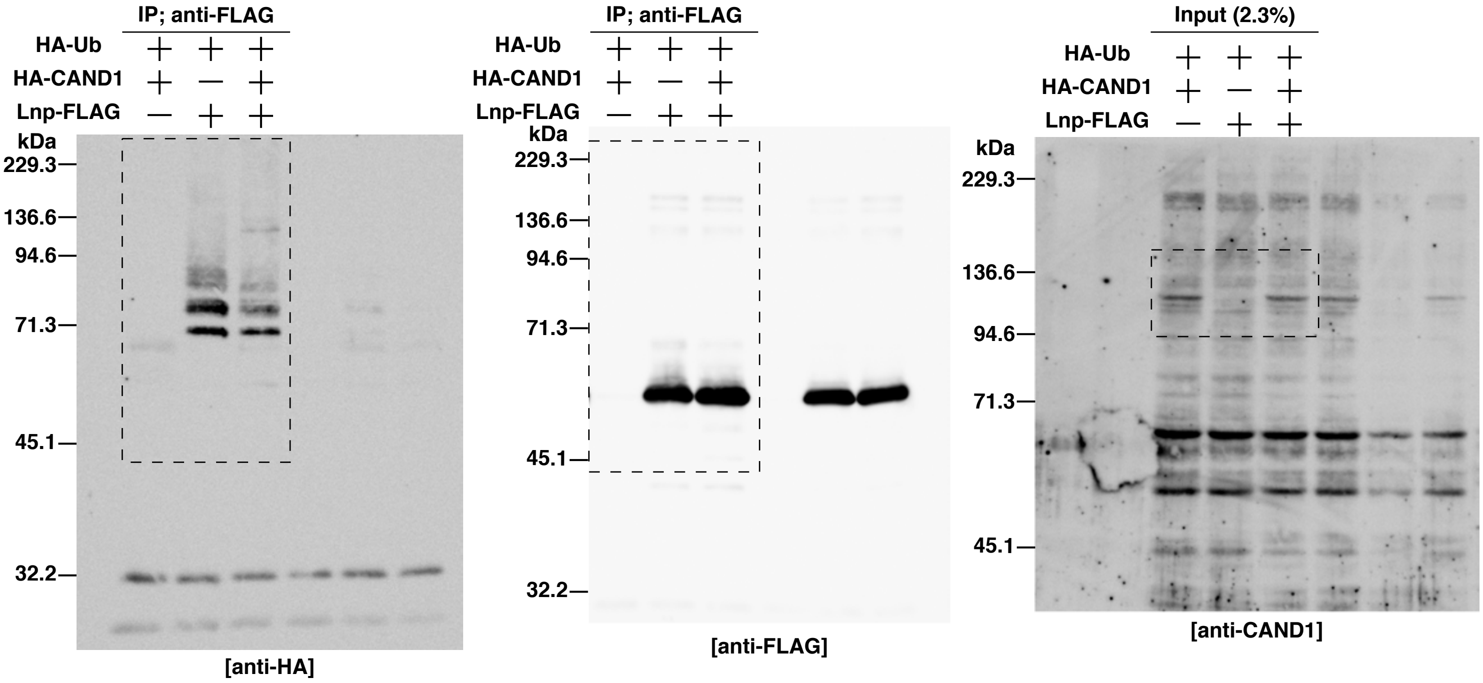

Supplementary figure S13

Figure 5a

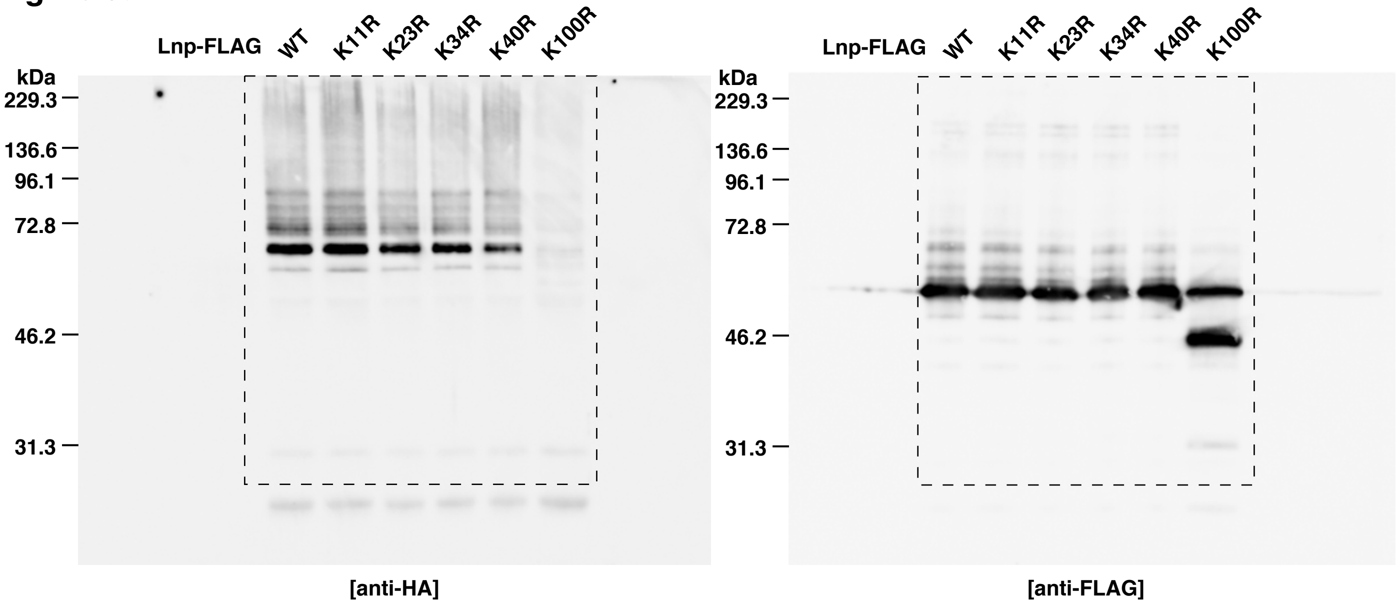

Figure 5b

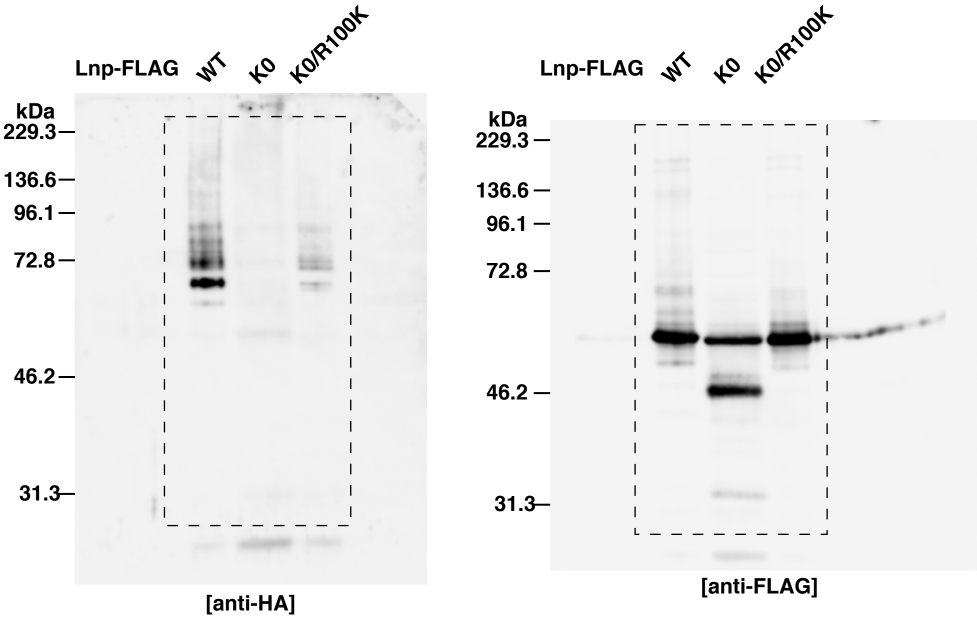

Supplementary figure S14

Figure 6a

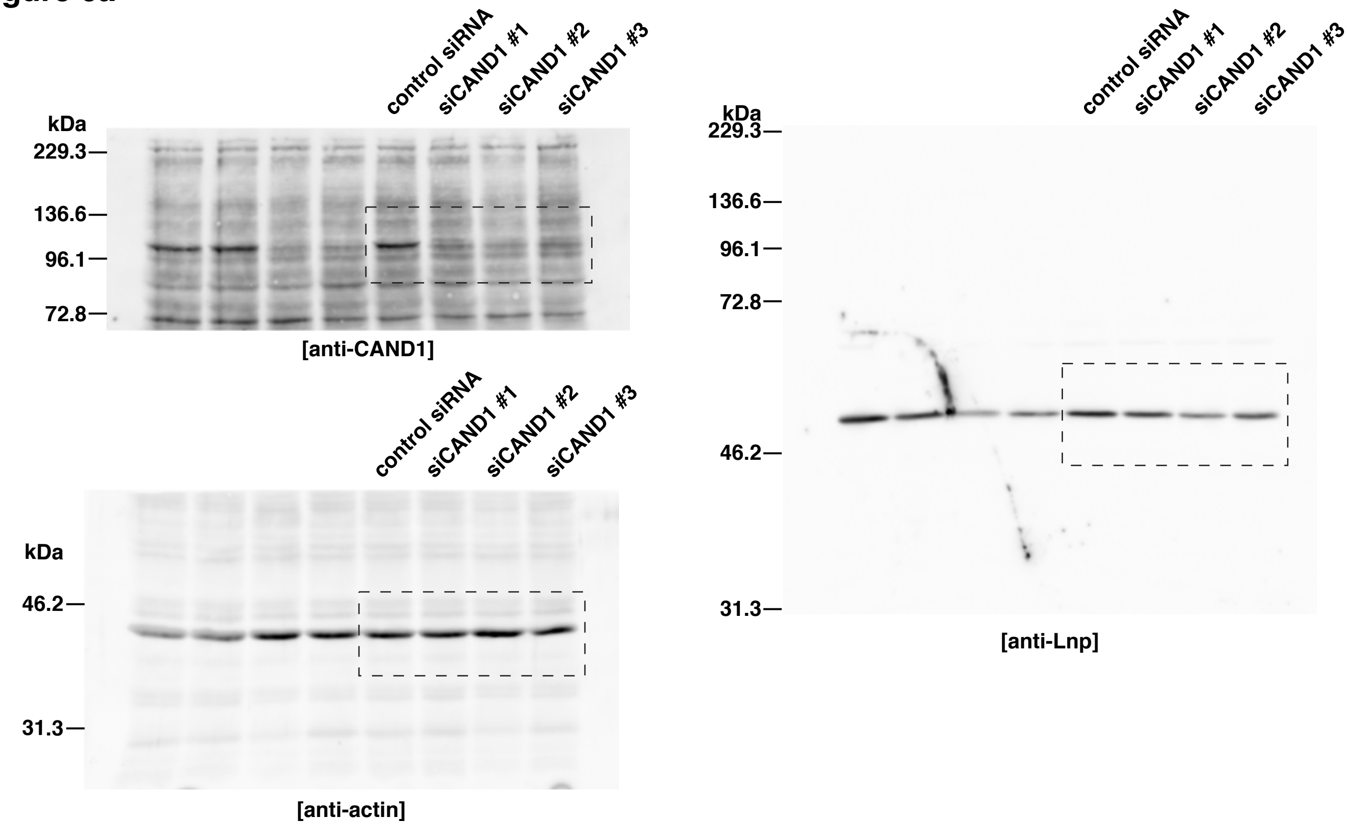

Figure 6b

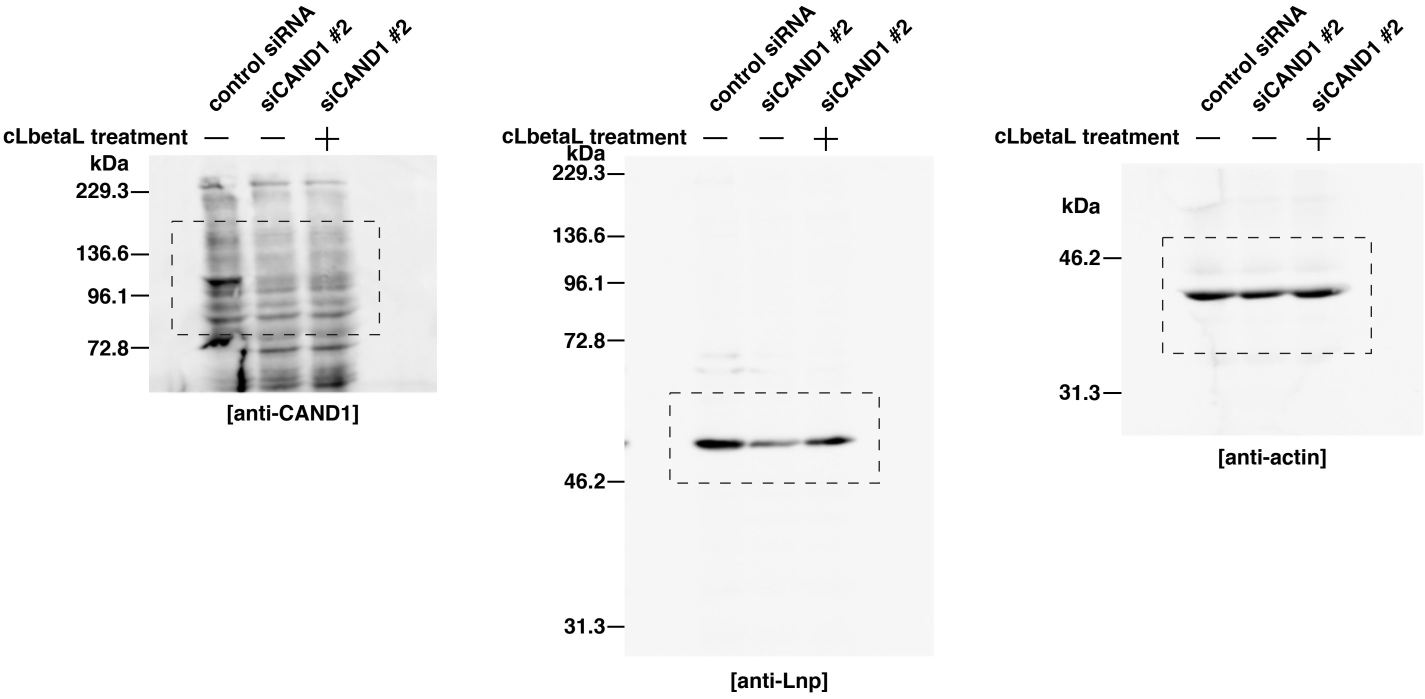

Supplementary figure S15

Supplementary figure S1

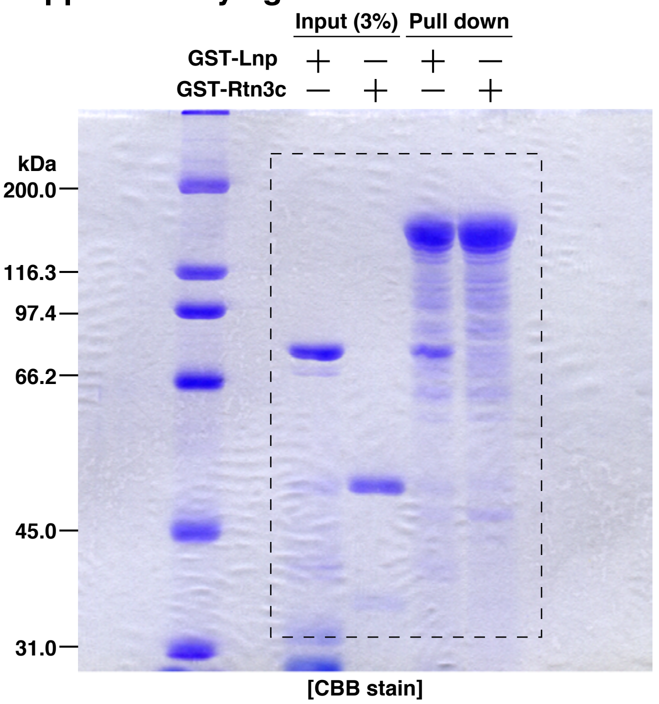

Supplementary figure S2b

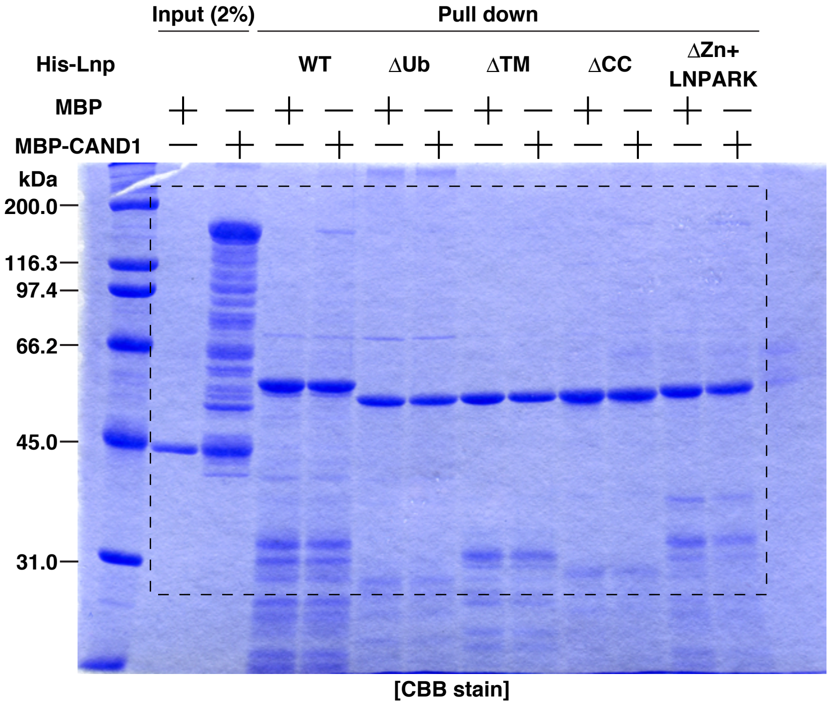

Supplementary figure S4a

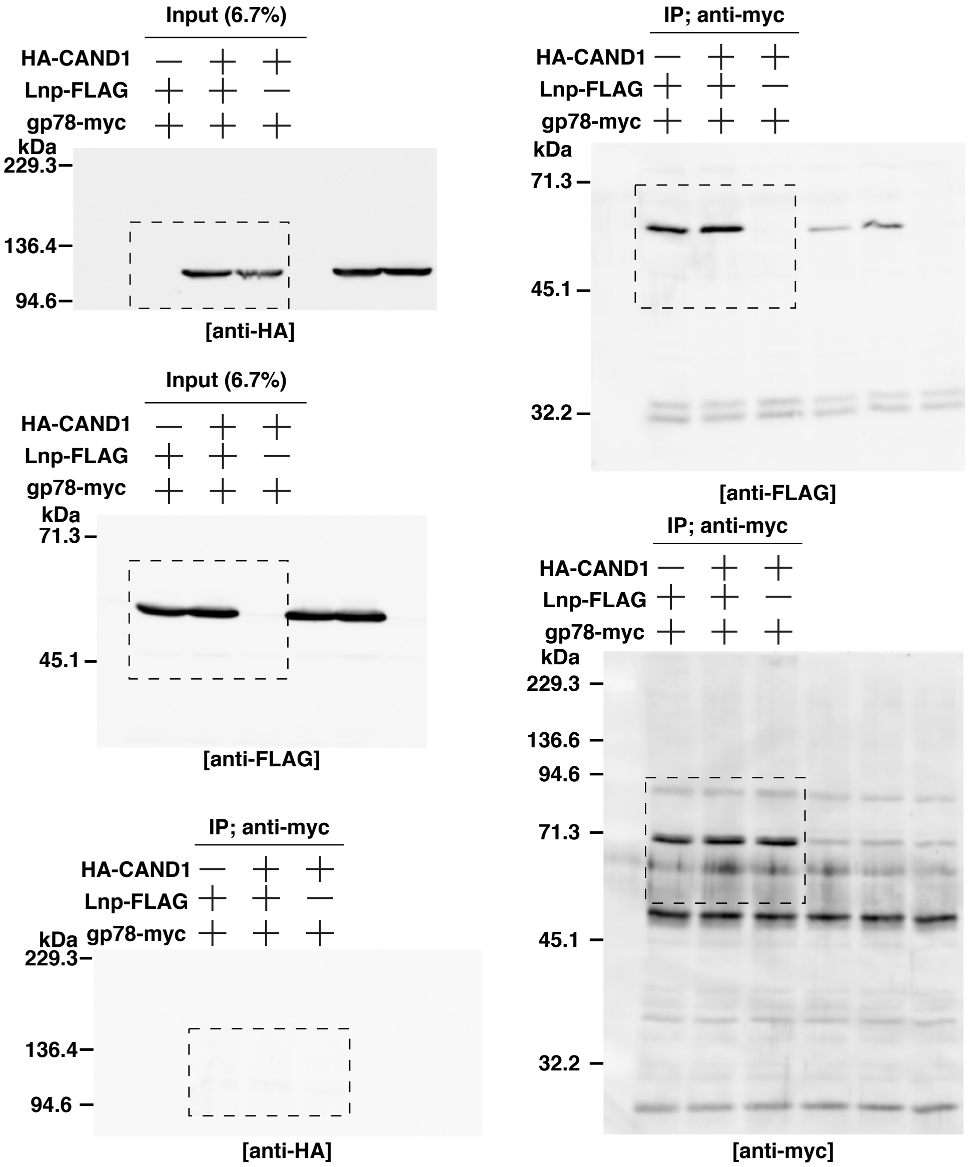

Supplementary figure S16

Supplementary figure S4b

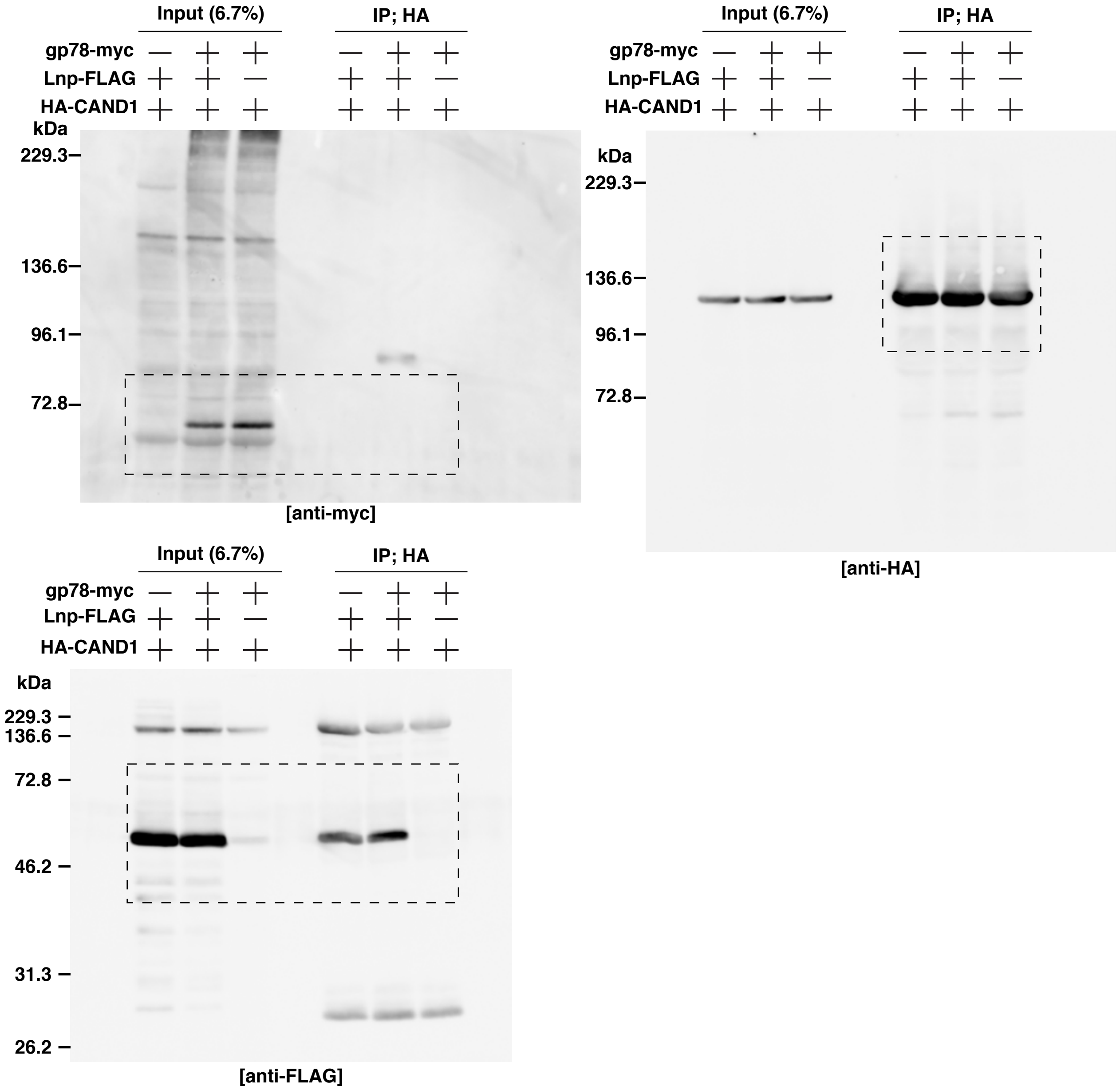

Supplementary figure S17

Supplementary figure S5a

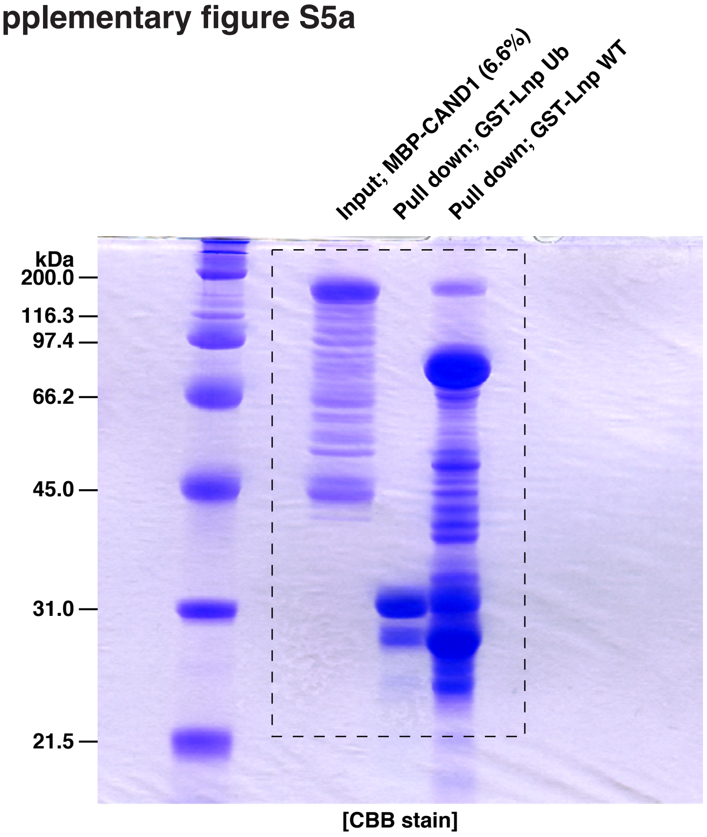

Supplementary figure S5b

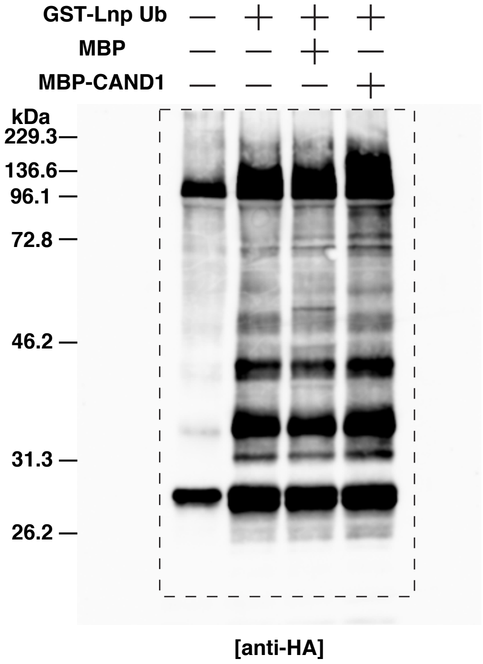

Supplementary figure S18

Supplementary figure S6b

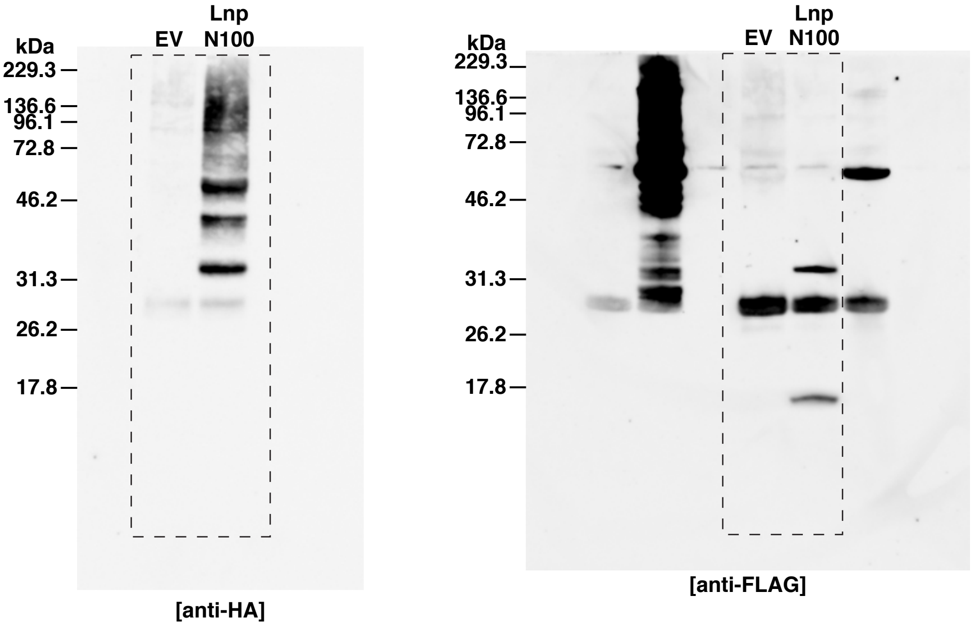

Supplementary figure S7

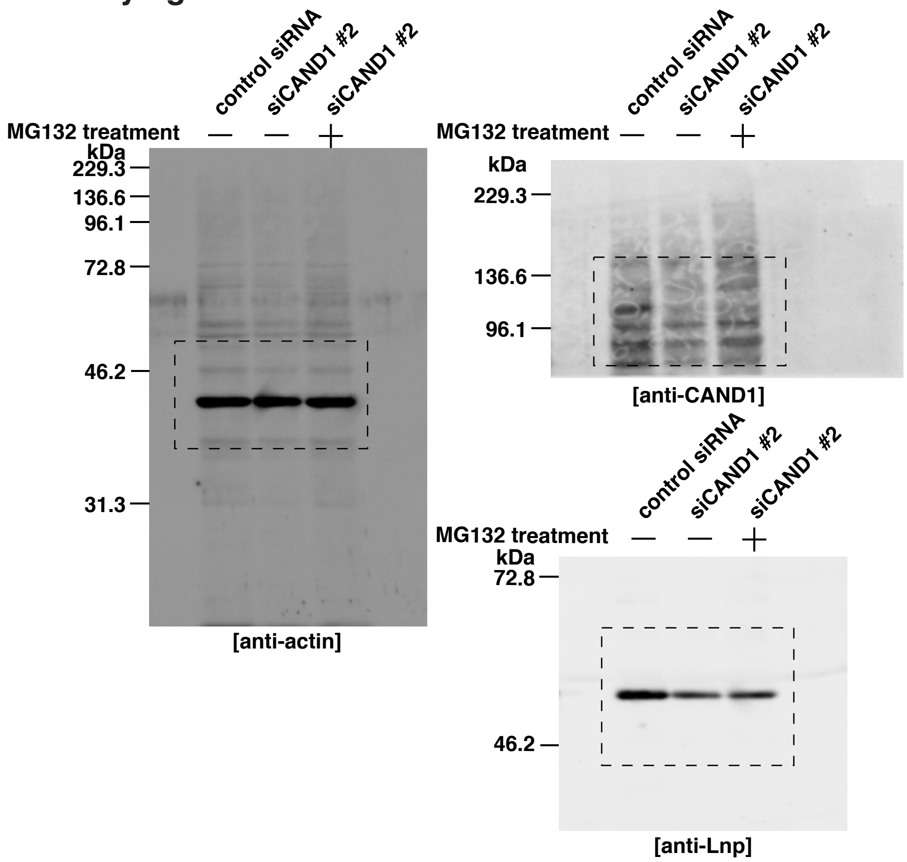

Supplementary figure S19

Supplementary figure S8

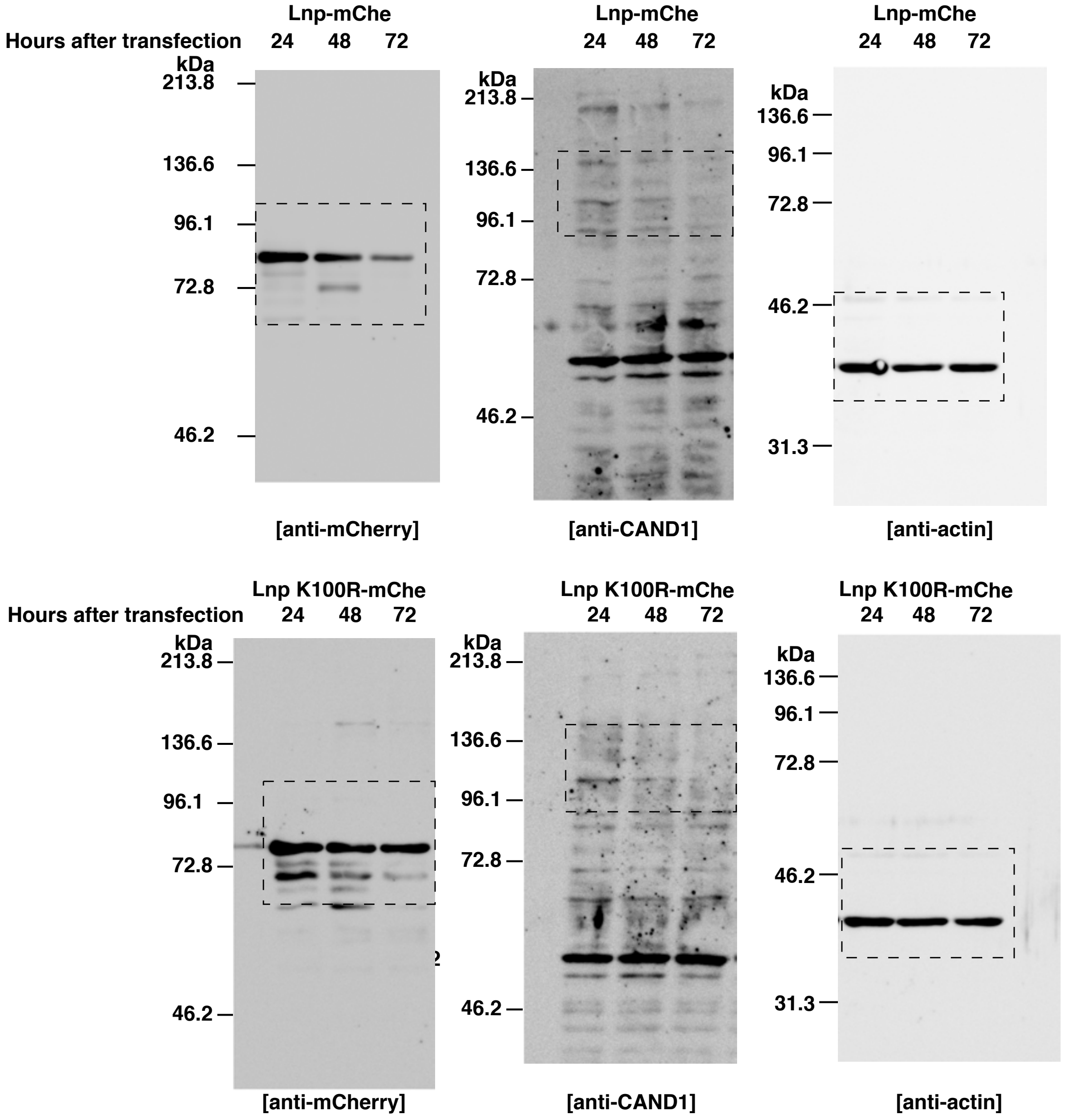

Supplementary figure S20
